# Checkpoint Inhibitor Pneumonitis Induced by Anti-PD-1/PD-L1 Therapy in Non-Small-Cell Lung Cancer: Occurrence and Mechanism

**DOI:** 10.3389/fimmu.2022.830631

**Published:** 2022-04-07

**Authors:** Jianqiong Yin, Yuanjun Wu, Xue Yang, Lu Gan, Jianxin Xue

**Affiliations:** ^1^ Department of Thoracic Oncology, Cancer Center and State Key Laboratory of Biotherapy, West China Hospital, Sichuan University, Chengdu, China; ^2^ Research Laboratory of Emergency Medicine, Department of Emergency Medicine, National Clinical Research Center for Geriatrics, West China Hospital, Sichuan University, Chengdu, China; ^3^ Department of Radiation Oncology, Cancer Center, West China Hospital, Sichuan University, Chengdu, China; ^4^ Laboratory of Clinical Cell Therapy, West China Hospital, Sichuan University, Chengdu, China

**Keywords:** programmed cell death 1, programmed cell death ligand 1, immune checkpoint inhibitors, immune-related adverse events, checkpoint inhibitor pneumonitis, non-small-cell lung cancer

## Abstract

Immune checkpointty inhibitors (ICIs), particularly those targeting programmed death 1 (PD-1) and anti-programmed death ligand 1 (PD-L1), enhance the antitumor effect by restoring the function of the inhibited effector T cells and produce durable responses in a large variety of metastatic and late patients with non-small-cell lung cancer. Although often well tolerated, the activation of the immune system results in side effects known as immune-related adverse events (irAEs), which can affect multiple organ systems, including the lungs. The occurrence of severe pulmonary irAEs, especially checkpoint inhibitor pneumonitis (CIP), is rare but has extremely high mortality and often overlaps with the respiratory symptoms and imaging of primary tumors. The development of CIP may be accompanied by radiation pneumonia and infectious pneumonia, leading to the simultaneous occurrence of a mixture of several types of inflammation in the lungs. However, there is a lack of authoritative diagnosis, grading criteria and clarified mechanisms of CIP. In this article, we review the incidence and median time to onset of CIP in patients with non-small-cell lung cancer treated with PD-1/PD-L1 blockade in clinical studies. We also summarize the clinical features, potential mechanisms, management and predictive biomarkers of CIP caused by PD-1/PD-L1 blockade in non-small-cell lung cancer treatment.

## 1 Introduction

Although the incidence and mortality of lung cancer has shown a significant decline according to recent studies, it still exceeds the vast majority of cancer types (1). According to the International Agency for Research on Cancer, lung cancer is the most common malignant tumor with the highest mortality. Non-small-cell lung cancer (NSCLC) is the major histological subtype of lung cancer, accounting for approximately 85% of all lung cancers (2). Anti-programmed death 1 (PD-1) and anti- programmed death ligand 1 (PD-L1) monoclonal antibodies (mAbs) have profoundly produced durable anticancer responses in patients with a variety of solid tumors, including NSCLC. The U.S. Food and Drug Administration has approved three PD-1 inhibitors (nivolumab, pembrolizumab and cemiplimab) and two PD-L1 inhibitors (atezolizumab and durvalumab) for the treatment of NSCLC with different stages (3–6).

However, accompanied by a promising survival advantage, PD-1/PD-L1 inhibitors are related to a broad spectrum of toxic effects known as immune-related adverse events (irAEs), including skin rash, colitis, hepatitis, endocrinopathies, and pneumonitis (7). In clinical trials, adverse events are reported and graded according to the Common Terminology Criteria for Adverse Events (CTCAE) from the U.S. National Cancer Institute, and immune-related pulmonary adverse events are graded from grade 1 to grade 5 depending on the symptoms from mild to serious (8). Most irAEs are mild and tolerable, while some of them can be fatal. In recent years, pulmonary adverse events caused by PD-1/PD-L1 inhibitors have been gradually reported, and some rare severe events even lead to death (9). It is worth noting that checkpoint inhibitor pneumonitis (CIP) accounts for 35% of PD-1 and PD-L1 inhibitor-related deaths (9). In addition, the incidence of CIP induced by anti-PD-1/PD-L1 is higher in patients with NSCLC than in patients with other cancers (3, 10). Pneumonitis can be mediated by various factors, such as radiotherapy, chemotherapy, targeted therapy, infection, and nonanticancer drugs (3). Thus, the diagnosis of CIP needs to be distinguished from hyperprogression, pseudoprogression, and other types of pneumonia (11). Further characterization of the unique clinical and radiographic features is needed to aid in the diagnosis of CIP. The incidence, risk, clinical characteristics and mechanism of CIP for anti-PD-1/PD-L1 monotherapy or a combination with other therapies (radiotherapy, chemotherapy or targeted therapy) may differ, which might influence the subsequent treatment options and prognosis of CIP (12). The clinical features and mechanisms of CIP induced by anti-PD-1/PD-L1 therapy in non-small-cell lung cancer have not been fully elaborated. In this review, we summarize recent discoveries on CIP introduced by anti-PD-1/PD-L1 treatment, especially in patients with NSCLC, and aim to provide a reference for clinical diagnosis and preclinical mechanistic research. We will also elucidate the incidence, time to onset and characteristic manifestations of CIP, as well as discuss the management and predictive biomarkers of CIP.

## 2 Occurrence of CIP During PD-1/PD-L1 Blockade

### 2.1 Incidence and Timeline

Pulmonary irAEs are more common in patients with NSCLC than in patients with other cancers, with an incidence of 3% to 5% according to clinical trial data (3, 10, 13). Without the exclusion criteria of clinical trials, the incidence of CIP can be much higher in the real world. In a retrospective study of 205 patients with NSCLC, 19% of them encountered CIP during PD-1/PD-L1 blockade. PD-1/PD-L1 inhibitors show a higher incidence of CIP than other ICIs, including cytotoxic T-lymphocyte-associated protein 4 (CTLA-4) inhibitors, and are responsible for 35% of CIP-related deaths (9, 14). The incidence of CIP can vary when different PD-1/PD-L1 inhibitors are used (4, 10, 15–27) (**Table 1**). However, in general, PD-1 inhibitors account for a higher incidence of all-grade (3.6% vs. 1.3%) and high-grade (1.1% vs. 0.4%) CIP than PD-L1 inhibitors based on a clinical trial of NSCLC (28). A multicenter, open-label, global, phase 3 trial compared the effect and safety of cemiplimab single-agent therapy with platinum-doublet chemotherapy in the first-line treatment of advanced NSCLC patients with a PD-L1 expression level of 50% or higher. The results showed that the incidence of immune-related pneumonitis in NSCLC patients receiving cemiplimab therapy was 2.3% (22).

**Table 1:** Important clinical trials that reported checkpoint inhibitor pneumonitis (CIP) in NSCLC patients with PD-1/PD-L1 blockade.

Registration number	Published year	Treatment	Enrollment ^a^	Phage	NSCLC stage	Incidence, n (%)		Reference
						Any grade	High grade (≥3)	
PD-1 inhibitor								
NCT01642004	2015	Nivolumab	131	III	IIIB or IV	6 (5.0)	1 (1.0)	(15)
NCT01673867	2015	Nivolumab	287	III	IIIB or IV	8 (2.8)	3 (1.0)	(16)
NCT02477826	2018	Nivolumab	391	III	IV	9(2.3)	6 (1.5)	(17)
NCT02477826	2018	Nivolumab + Ipilimumab	576	III	IV	22 (3.8)	13 (2.3)	(17)
NCT01295827	2015	Pembrolizumab	550	III	IIIB or IV	21 (3.8)	11 (2.0)	(18)
NCT01905657	2016	Pembrolizumab	682	II/III	IIIB or IV	26 (3.8)	12 (1.8)	(19)
NCT02142738	2016	Pembrolizumab	154	III	IIIB or IV	9 (5.8)	4 (2.6)	(20)
NCT02220894	2019	Pembrolizumab	636	III	IIIB or IV	53 (8.3)	22 (3.5)	(21)
NCT02775435	2018	Pembrolizumab + Carboplatin+ (Nab-) Paclitaxel	278	III	IV	18 (6.5)	7 (2.5)	(10)
NCT03088540	2021	Cemiplimab	355	III	IIIB, IIIC or IV	8 (2.3)^b^	2 (0.6)^c^	(22)
PD-L1 inhibitor								
NCT02008227	2017	Atezolizumab	609	III	IIIB or IV	6 (1.0)	4 (0.7)	(4)
NCT02409342	2020	Atezolizumab	286	III	IV	11 (3.8)	2 (0.7)	(23)
NCT02657434	2021	Atezolizumab + Pemetrexed	291	III	IV	18 (6.2)	6 (2.1)	(24)
NCT02367781	2019	Atezolizumab + Carboplatin + Nab-paclitaxel	473	III	IV	25 (5.3)	2 (0.4)	(25)
NCT02125461	2017	Durvalumab	475	III	III	51 (10.7)	8 (1.7)	(26)
NCT02453282	2020	Durvalumab	369	III	IV	8 (2.2)	5 (1.4)	(27)
NCT02453282	2020	Durvalumab + Tremelimumab	371	III	IV	25 (6.7)	11 (3.0)	(27)

NSCLC, non-small-cell lung cancer; PD-1, programmed cell death 1; PD-L1, programmed cell death ligand 1.

^a^Patients enrolled in and received ICI treatment.

^b^Includes 7 immune-related pneumonitis and 1 immune-mediated pneumonitis.

^c^Includes 1 immune-related pneumonitis and 1 immune-mediated pneumonitis.

There is a lower incidence of pneumonitis in PD-1/PD-L1 inhibitor monotherapy than in concurrent or sequential chemotherapy (29), radiotherapy (30) or immunotherapy (3, 17, 27, 31). In a randomized phase III clinical trial, the incidences of any-grade and high-grade CIP in NSCLC patients receiving durvalumab plus tremelimumab, a CTLA-4 inhibitor, were higher than those with durvalumab monotherapy (6.7% vs. 2.2% for any-grade CIP and 2.2% vs. 1.1% for high-grade CIP) (27). This may be attributed to the overlapping pulmonary toxicity of different treatments. In addition, there are no reliable criteria to distinguish CIP from radiation pneumonitis and radiation recall pneumonitis, which may affect the diagnosis of CIP (32, 33). Concurrent or sequential combination therapy is common in the clinic and may account for the higher incidence of treatment-related pneumonitis in the real world (34–36).

**Figure 1:**
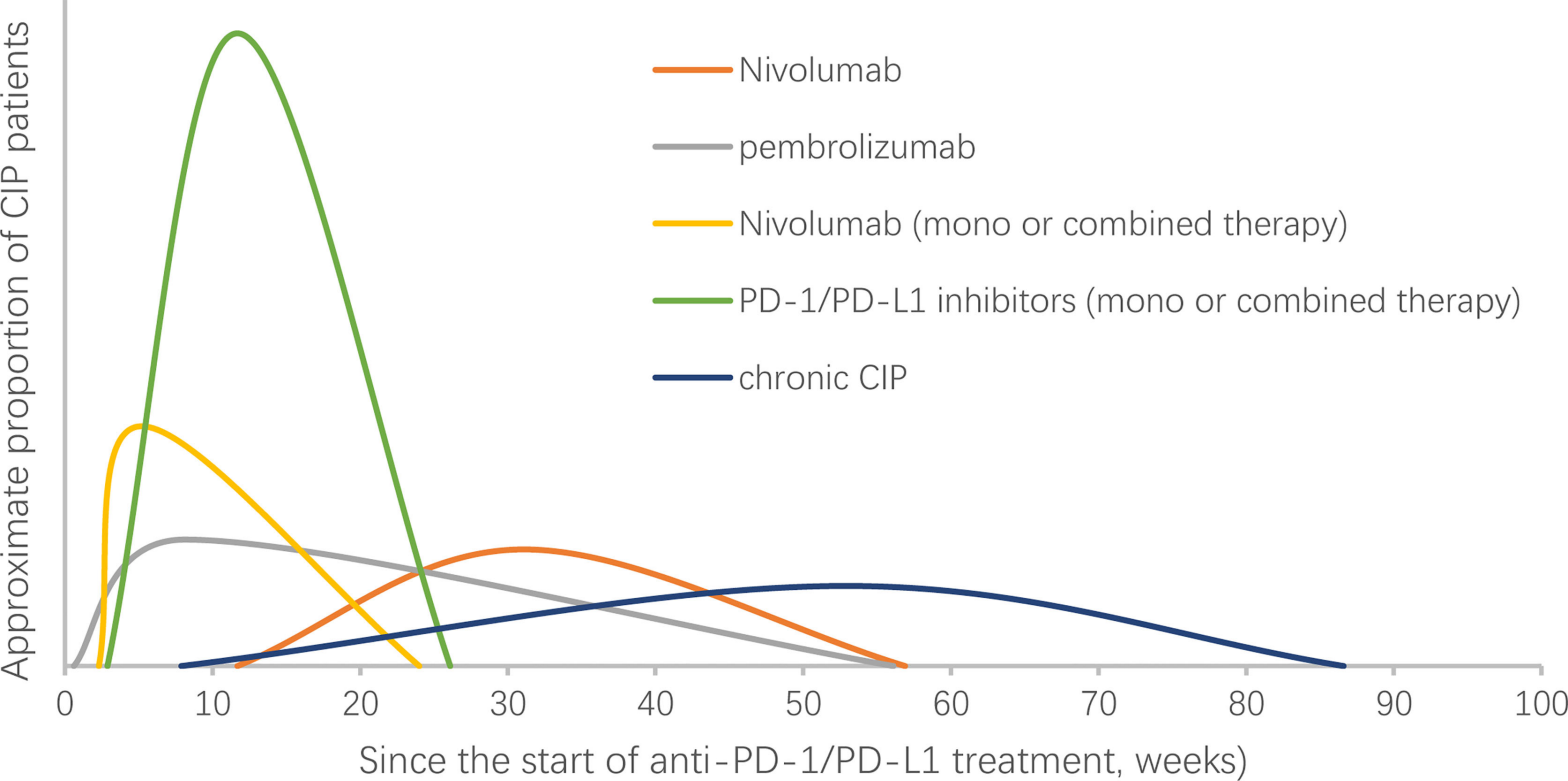
Onset time of checkpoint inhibitor pneumonitis (CIP) in NSCLC patients receiving PD-1/PD-L1 treatment. NSCLC, non-small-cell lung cancer. The curve in the figure does not represent the change in the incidence of CIP over time. The abscissa of the highest point of the curve represents the median time of CIP reported in the studies (16, 18, 34, 35, 37).

The median time to onset of pulmonary adverse events can vary. A report showed that the median time to onset of pneumonitis was 2.8 months, with a wide range from 9 days to 19.2 months (3). The onset time may be affected by the selection of the ICI agent (15, 16, 18, 34–37) (**Table 2**). In a randomized, open-label, international phase 3 study, the median onset time to pneumonitis was 15.1 (2.6-85.1) weeks and 31.1 (11.7-56.9) weeks in patients with advanced squamous and nonsquamous NSCLC who received nivolumab, respectively (15, 16). In another randomized controlled trial, the median onset time was 8.1 (0.6-56.1) weeks in advanced NSCLC patients treated with pembrolizumab (18). The treatment strategy may also influence the onset time. A shorter median onset time was reported in patients with combination immunotherapy compared with monotherapy (2.7 months (9 days to 6.9 months) vs. 4.6 months (21 days-19.2 months), P=0.02) (3). In retrospective studies that enrolled only NSCLC patients, the onset of CIP occurred earlier when PD-1/PD-L1 inhibitors were combined with other treatments (**Table 2**). More high-grade CIP onset occurs within the first 100 to 200 days of immunotherapy (38). Naidoo et al. reported a type of CIP called chronic CIP, which refers to a kind of clinical pneumonitis persisting or worsening with steroid tapering and necessitating ≥12 weeks of immune suppression after ICI discontinuation. The incidence of chronic pneumonitis is approximately 2.4% in patients with NSCLC, and the onset time of chronic CIP is variable (range: 238–606 months) and occurs at a median time of 370 months after ICI start (37). In summary, the onset time of CIP shows various timespans and may be affected by many factors, including treatment regime (**Figure 1**).

**Table 2:** Study that reported the onset time of checkpoint inhibitor pneumonitis (CIP) in NSCLC patients with PD-1/PD-L1 blockade.

Published Year	Treatment	Study type	Enrollment ^a^	NSCLC stage	Incidence, n (%)		Median time to onset, week(range)	Reference
					Any grade	High grade (≥3)		
2015	Nivolumab	Prospective, RCT	131	III	6 (5.0)	1 (1.0)	15.1 (2.6–85.1)	(15)
2015	Nivolumab	Prospective, RCT	287	III	10 (3.5) ^b1^	4 (1.4) ^b2^	31.1 (11.7-56.9)	(16)
2015	Pembrolizumab	Prospective, RCT	550	IIIB or IV	21 (3.8)	11 (2.0)	8.1 (0.6-56.1)	(18)
2017	Nivolumab (mono or combined with chemotherapy)	Retrospective	111	IV or recurrent	8 (7.2) ^b3^	4 (3.6) ^b3^	5.2 (2.3–24)	(34)
2018	Nivolumab/Pembrolizumab/Durvalumab (mono or combined with other ICI/chemotherapy)	Retrospective	205	all	39 (19.0)	24 (11.7)	11.7 (2.9–26.1) ^c^	(35)
2020	Nivolumab (mono or combined with chemotherapy)	Retrospective	901	all	94 (10.4)	39 (4.3)	8.4 (0-75.1)	(36)
2020	Nivolumab/Ipilimumab/Pembrolizumab (mono or combined with chemotherapy)	Retrospective ^d^	205	all	5 (2.4) ^e^	2 (1.0) ^e^	52.9 (34-86.6)	(37)

NSCLC, non-small-cell lung cancer; PD-1, programmed cell death 1; PD-L1, programmed cell death ligand 1.

^a^Patients enrolled in and received ICI treatment.

^b^Includes patients with interstitial lung disease (2 in a1, 1 in a2, all in a3).

^c^Interquartile range of onset time.

^d^A retrospective study on Chronic pneumonitis (clinical pneumonitis persisting or worsening with steroid tapering and necessitating ≥12 weeks of immune suppression after ICI discontinuation).

^e^Initial pneumonitis grade.

### 2.2 Risk Factors

Checkpoint inhibitor pneumonitis may be the preferred attack in patients with features of lung deterioration conditions, including aging, smoking status, prior treatment, combinations with other drugs, primary tumor type, and previous lung disease. A previous study found that patients aged >70 years were more common in a CIP group than in a non-CIP group (54.5% vs. 30.3%; P = 0.025) (39), which may be due to decreased lung function and increased medical complications in the elderly. In a study by Suresh et al. (35), a higher incidence of CIP was observed in males than in females and in squamous cells than in other histological types. Notably, smoking may also play a role in the development of CIP. Former/current smokers were found to have a higher incidence of pneumonitis than nonsmokers (P=0.03) (40). The incidence of CIP is also influenced by treatment strategy. Currently, a significant number of patients with locally advanced unresectable or metastatic NSCLC receive treatment with radiotherapy alone or concurrent chemotherapy before receiving immunotherapy (41). Multiple clinical studies have shown that the incidence of CIP was numerically higher in patients receiving chest radiotherapy than in those receiving nonchest/no radiotherapy (40, 42–45). Some researchers consider this kind of pneumonitis radiation recall pneumonitis (RRP) introduced by immunotherapy, while the criteria for identifying CIP and RRP are still unclarified (33). However, radiation parameters did not correlate with the development of pneumonitis (40). As mentioned above, PD-1 inhibitors are related to a higher incidence of CIP both for any grade or ≥ grade 3 compared with PD-L1 inhibitors. PD-1/PD-L1 blockades are usually combined with chemotherapeutic drugs, tyrosine kinase inhibitors, or additional immune-targeted drugs. The combination of PD-1/PD-L1 inhibitors with a variety of different drugs was also associated with pneumonitis risk, indicating the superposition effect of multiple drugs on pulmonary toxicity (10, 17, 31, 45, 46). Patients with epidermal growth factor receptor (EGFR) mutations are more likely to undergo pneumonitis during the combination therapy of sequential PD-(L)1 blockade followed by later osimertinib (47). The risk of CIP was also closely associated with preexisting lung disease, including pulmonary infection (48), pulmonary emphysema (49), chronic obstructive pulmonary disease (COPD) (50), asthma (50), interstitial lung disease (ILD) (51), pulmonary fibrosis (52), pneumothorax, and pleural effusion (45). Interestingly, a recent study found that tumor invasion into the central airway was strongly associated with early-onset CIP in patients with NSCLC (53). The presence of extrathoracic metastasis was related to a lower incidence of ICI-related pneumonitis (39). In addition, according to a recent study, a high baseline absolute eosinophil count (≥0.125 ×10^9^ cells/L) was correlated with an increased risk of CIP and with a better clinical outcome (54). A higher baseline level of anti-CD74 autoantibody is also more likely to develop CIP (55). Taken together, the risk factors for CIP may include older age, history of smoking, squamous cell histological type, previous lung disease, prior thoracic irradiation and treatment combinations with other drugs (**Table 3**).

**Table 3:** Potential risk factors for checkpoint inhibitor pneumonitis (CIP) in NSCLC.

Potential risk factors	Details
Sex	Males have a higher incidence of CIP
Age of patients	**·**Patients aged >70 years
	**·**The decreased lung function and increased medical complications in the elderly may be the cause of higher incidence of CIP
Tumor histologic type	A higher incidence of CIP in patients with squamous NSCLC
Smoking status	Patients with former or current smoking
Prior thoracic	The incidence of CIP is numerically higher in patients receiving chest-RT compared with non-chest/no RT
radiation therapy	**·**Radiation parameters have no correlation with the development of pneumonitis
	**·**The influence of radiotherapy courses, type and timing for development of CIP is still not clear
	**·**T cells activated during ICIs treatment are more easily infiltrate into the damaged lung tissue by thoracic irradiation
PD-1 inhibitors	**·**The incidence of CIP with PD-1 inhibitors is higher than PD-L1 inhibitors
	**·**PD-1/PD-L1 inhibitors are related to a higher incidence of CIP than anti-CTLA4
	**·**PD-1 inhibitors lead to increased risk through increasing the interaction of PD-L2- RGMb
Combination therapy	**·**Additional immune-targeted drugs, chemotherapeutic drugs and some specific tyrosine kinase inhibitors (TKIs)
	**·**Combination of PD-1/PD-L1 inhibitors with other antitumor agents might mediate the superposition effect of multiple drugs on pulmonary toxicity
EGFR mutation	Patients with EGFR mutation are more likely to undergo pneumonitis during the combination therapy of ICIs with osimertinib
Sequences of drug administration	PD-(L)1 blockade followed by osimertinib is related to a higher incidence of pneumonitis
Preexisting lung disease	Pulmonary infection, pulmonary emphysema, COPD, asthma, ILD, pulmonary fibrosis, pneumothorax, and pleural effusion
Tumor invasion	**·**Tumor invades the central airway was strongly associated with early-onset CIP
	**·**Extrathoracic metastasis was related to a lower incidence of CIP
Baseline peripheral-blood absolute eosinophil count (AEC)	A high level of baseline AEC(≥0.125×10^9^cells/L) correlated with an increasing risk of CIP but a better clinical outcome
Baseline level of anti-CD74 autoantibody	A higher baseline level of anti-CD74 autoantibody is also more likely to develop CIP

NSCLC, non-small-cell lung cancer; CIP, checkpoint inhibitor pneumonitis; RT, radiation therapy; PD-1, programmed cell death protein 1; PD-L1, programmed cell death protein ligand-1; CTLA-4, cytotoxic T lymphocyte-associated protein 4; PD-L2, programmed cell death protein ligand-2; RGMb, repulsive guidance molecule b; TKIs, tyrosine kinase inhibitors; EGFR, epidermal growth factor receptor; COPD, chronic obstructive pulmonary disease; ILD, interstitial lung disease; AEC, absolute eosinophil count.

### 2.3 Effects of CIP on the Clinical Outcome of Patients With PD-1/PD-L1 Therapy

Several studies have indicated that melanoma patients with irAEs have a survival benefit (56–59), which suggests that the early occurrence of irAEs may predict better outcome of immune checkpoint inhibitor therapy, and appropriate management of these events is needed to maximize the therapeutic effect of these drugs. The mechanisms behind this association are not fully understood. It has been proposed that the shared antigens between melanoma cells and normal melanocytes may account for this association (57, 59, 60). Similarly, several studies have shown favorable treatment efficacy and survival in patients with NSCLC (2, 61, 62). However, whether there is a similar association between CIP and tumor response in patients with NSCLC remains controversial. Genova et al (62) have shown that the development of irAEs, including CIP, is a significantly positive predictor of survival outcomes in patients with NSCLC treated with nivolumab. However, in another study, the efficacy of ICI treatment was observably worse in patients with severe grade CIP than in those without severe grade CIP when patients were classified according to CIP severity (63). Thus, several challenges still need to be addressed in the future, and the association between the development of CIP and clinical efficacy in patients with NSCLC treated with ICIs still needs to be evaluated. In addition, although studies have shown that the occurrence of CIP is associated with a better response to ICI therapy, severe CIP can lead to interruption of ICI therapy and even fatality. In conclusion, the effects of CIP on the efficacy and prognosis of ICI therapy in NSCLC patients are still controversial, and more evidence is needed to clarify the relationship.

### 2.4 Overview of Manifestations

#### 2.4.1 Common Symptoms

The most common clinical symptoms of CIP are dyspnea and cough. Fever and chest pain can also be observed but not often (3). However, it is difficult to distinguish these symptoms from infection or progression of malignancy, especially in patients with NSCLC. For individuals with suspected CIP, laboratory examinations are necessary to exclude infection, including culture of nasopharyngeal, sputum, and urine and sensitivity tests. In addition, some patients with subclinical CIP may experience no respiratory symptoms. Therefore, the diagnosis of CIP also depends on other auxiliary examinations (64).

#### 2.4.2 Radiological Features

Radiological examination, especially chest CT, plays an important role in the diagnosis of CIP. Several radiographic patterns of CIP have been observed, including cryptogenic organizing pneumonia (COP), nonspecific interstitial pneumonia, hypersensitivity pneumonitis and pneumonitis not otherwise specified (3). The most predominant radiographic pattern is COP (65). Specific findings on chest CT include traction bronchiectasis, consolidation, reticular opacities, ground glass opacities (GGOs), centrilobular nodularity, and honeycombing, of which GGOs are present in the majority of patients (66). In addition, GGO was indicated as a significant predictor of worse overall survival (67). Clinically, the situation will be more complicated, and mixed patterns may be observed. In a case of a 73-year-old woman with NSCLC who received pembrolizumab treatment, organizing pneumonia with “air bronchogram” presented at the upper lung lobes and widespread thickening of the interlobular interstitium at the left lower lobe (68). The use of PET-CT in the diagnosis of immune-related pneumonia in patients with melanoma has been reported, but there were few specificities of the radiological images of pneumonia on PET/CT scans (69, 70). As mentioned above, there are several radiological patterns of CIP, and detailed analysis is needed when facing different patterns of CIP in the clinic.

#### 2.4.3 Auxiliary Examinations

Bronchoalveolar lavage (BAL) is one of the most common invasive examinations of ICI-related pneumonitis. In general, lymphocytes elevated in bronchoalveolar lavage fluid (BALF). Nandoo et al. (37) found that lymphocytes, predominantly CD4^+^ T cells, were increased in the BALF of CIP patients compared with the BALF from patients who received ICI treatment but had no evidence of CIP (n= 6) or suspected CIP patients (n= 14). Wang et al. found significantly elevated levels of interleukin-17A (IL-17A) and IL-35 in both serum and BALF (71). In addition, they also observed increased numbers of central memory T cells and decreased expression of CTLA-4 and PD-1 in regulatory T cells (Tregs). Enriched IFNg^+^IL-17-CD8^+^T and CXCR3^+^CCR6^+^Th17/Th1 cells were observed in the BALF of CIP compared with infection pneumonia in acute myeloid leukemia and myelodysplastic syndrome (67).

Transbronchial biopsy is seldom used in the auxiliary diagnosis of CIP. In the few cases of transbronchial biopsy, pneumonia tissue after ICI showed inflammation and lymphocyte infiltration (39). Nandoo et al. collected 11 tissue samples by transbronchial biopsy (8/11), core biopsy (2/11) and wedge resection (1/11), and the histopathological results included 4 cellular interstitial pneumonitis, 3 organizing pneumonia, 1 diffuse alveolar damage and 3 no abnormalities identified (3).

## 3 Mechanisms of PD-1/PD-L1 Inhibitor-Induced Pulmonary Toxicity

**Figure 2:**
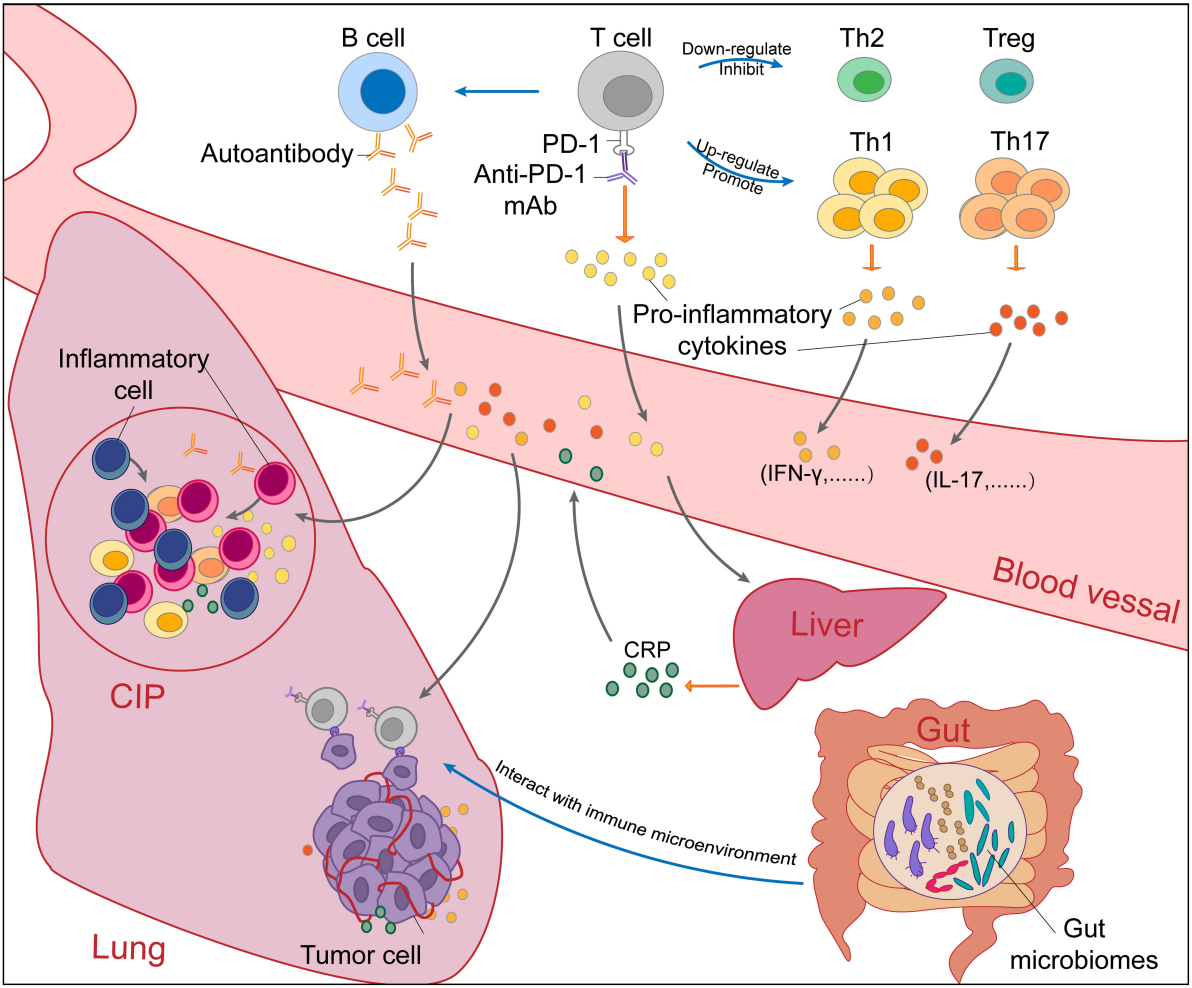
The potential mechanisms of checkpoint inhibitor pneumonitis (CIP) in NSCLC patients receiving PD-1/PD-L1 inhibitor monotherapy. The occurrence of checkpoint inhibitor pneumonitis (CIP) in NSCLC is the result of a combination of many factors. Blockade of the PD-1-PD-L1 pathway by PD-1/PD-L1 mAbs (PD-1 mAb in the figure, for example) will upregulate and promote Th1 and Th17 cells and downregulate and inhibit Th2 cells and Tregs. Without immunosuppression of Th2 cells and Tregs, excessive immune responses and cytokine secretion of Th1 and Th17 cells will cause autoimmune damage in normal tissues such as the lung. In addition, autoantibodies increased after PD-1/PD-L1 blockade can also cause normal tissue lesions. Proinflammatory cytokines secreted by activated T cells promote the infiltration of inflammatory cells. Under the stimulation of IL-6, CRP produced by the liver will promote inflammation and strengthen autoimmunity. Through the “gut-lung axis”, gut microbiomes can regulate the immune microenvironment in the lung. Overall, the immune dysregulation caused by PD-1/PD-L1 blockade leads to the occurrence and development of CIP. NSCLC, non-small cell cancer; CIP, checkpoint inhibitor pneumonitis; PD-1, programmed cell death protein 1; PD-L1, programmed cell death protein ligand-1; mAbs, monoclonal antibody; Th, helper T cell; IL, Interleukin; CRP, C-reactive protein.

Due to the lack of effective preclinical studies, the mechanism of PD-1/PD-L1 inhibitor-induced CIP in NSCLC patients is still unclear. Here, we summarize the factors that may be involved in CIP based on existing studies (**Figure 2**).

### 3.1 Disordered T Cell Subsets

PD-1/PD-L1 inhibitors can enhance the antitumor function of T cells (72). There is some evidence that T cells may be involved in the development of immune-related pulmonary complications. Increased infiltration of highly proliferative CD8^+^ T cells was observed in lung biopsy tissue from patients with NSCLC who developed chronic bronchiolitis obliterans organizing pneumonia after nivolumab treatment (37). Suresh et al. found that CD4^+^ T cells were significantly increased in BAL samples of CIP patients (mainly with NSCLC) after receiving anti-PD-1/PD-L1 inhibitors (73). Suzuki et al. reported that the proportion of CD8^+^ T cells with PD-1, Tim-3 and TIGHIT positivity in the BALF of patients with PD-1/PD-L1 inhibitor-induced ILD was significantly higher than that of patients with other types of ILD (74). All of these findings suggest dysregulated alveolar immunity in CIP patients. Kim et al. found significant elevation of Ki-67 (proliferation markers of PD-1^+^ CD8^+^ T cell) in metastatic NSCLC patients who received pembrolizumab or nivolumab. Various studies have shown that T cells are activated and infiltrate the lung tissue of CIP patients, which indicates the enhancement of antitumor effects. However, an excessive immune response may lead to damage to normal tissue.

When naïve T cells encounter antigens presented by antigen-presenting cells (APCs) in secondary lymphoid organs, they undergo differentiation/polarization processes according to cell division signals and become T helper 1 (Th1) cells, T helper 2 (Th2) cells, T helper 17 (Th17) cells or Tregs (72). Different T cell subsets may play different roles in irAEs.

#### 3.1.1 Th1/Th2

A Th1/Th2 shift exists in tumor patients, and Th2 cells are often dominant (75). It has been suggested that the shift of Th1/Th2 cells may be related to the immune escape of tumors (76). Anti-PD-1/PD-L1 therapy can reverse Th1/Th2 in cancer patients by promoting the production of Th1 cells and inhibiting the production of Th2-related cytokines (77). However, Th1 cells may be the dominant response cell in the development of irAEs, as infiltration of Th1 cells has been observed in related tissues. Yoshino et al. reported nivolumab-related colitis in 2 patients with metastatic melanoma, whose pathological examinations of the colon showed infiltration of CD8^+^ T cells and T-bet-expressing Th1 CD4^+^ T cells (78). Kim et al. reported enrichment of T-bet ^+^ POPGT^+^ (Th1) and CXCR3 ^+^ T-bet ^+^ CCR6 ^+^ RORGT^+^ (Th17/Th1) cells in BAL CD4^+^ T cells of leukemia patients with respiratory symptoms after ICI-based therapy. Interestingly, most Th17/Th1 cells express PD-1 (79). These studies indicated that Th1 cells may be involved in the formation and development of anti-PD-1-associated pneumonitis. In addition, Th1 cells are also associated with some autoimmune diseases (80), which may explain autoimmune symptoms after PD-1/PD-L1 blockade.

#### 3.1.2 Treg

Tregs are an important factor in maintaining immune tolerance. Nedoszytko et al. reported that Tregs with high activity can lead to immunosuppression and decrease the number of Th1 cells (81). Tregs express both PD-1 and PD-L1 (82). Amarnath et al. found that human Th1 cells transform into Tregs through the involvement of the PD-1/PD-L1 axis (83). Francisco et al. reported that PD-L1 plays an important role in converting naïve CD4^+^ T cells into induced Tregs (iTregs) as well as maintaining and strengthening their immunosuppressive function (82). When blocking the PD-1/PD-L1 axis, differentiation to Tregs may be prevented, and a decreased number of Tregs can be observed in the tumor microenvironment (TME) (84). This suggests that anti-PD-1/PD-L1 treatment could be a potential strategy to improve the anti-infective and antitumor immunity of T cells. However, immune-related damage occurs at the same time. In Francisco’s study, fatal immune-mediated pulmonary damage and iTreg differentiation were observed *in vivo* in PD-L1^−/−^PD-L2^−/−^Rag^−/−^recipients of naïve CD4^+^T and Rag^−/−^mice treated with a PD-L1 inhibitor (82). In addition, Suresh et al. found decreased expression of CTLA-4 and PD-1 on Tregs in the BALF of CIP patients, indicating the loss of the inhibitory phenotype of Tregs (73). Therefore, the lack and poor function of Tregs may lead to unchecked immune dysregulation, which may lead to irAEs such as CIP.

#### 3.1.3 Th17

Th17 cells are a subset of T cells that produce IL-17. Th17 lymphocytes exist in the anatomic barrier, mainly in the digestive system and lungs (85). In previous studies, the antitumor role of Th17 cells seems to be contradictory. On the one hand, Th17 cells can recruit CD8^+^ cytotoxic T cells and promote their activation and expansion to inhibit the growth of tumors (86, 87). On the other hand, IL-17A produced by Th17 cells has been shown to enhance tumor angiogenesis (88). Jaclyn W. McAlees et al. found that the levels of Th1 and Th17 cells increased in naïve PD-1^−/−^ mice, while the production of cytokines in polarized Th1 and Th17 cells *in vitro* was restricted in WT cells with PD-1 ligation (89). In this way, anti-PD-1 therapy may enhance the antitumor function of Th17 cells. Yun et al. found that under certain conditions, Th17 cells can transform into Th1 cells, lose the secretion of IL-17A, and then secrete interferon γ (IFN-γ), which plays a role in enhancing autoimmunity and antitumor activity (90). As mentioned above, Tregs can suppress the amplification of Th1 cells, but Tregs cannot inhibit the transformation of Th17 cells into Th1 cells (91). After blocking PD-1/PD-L1, the decrease in Tregs may lead to an imbalance in Treg/Th17 cells. The dysregulation of Treg/Th17 cells is related to a variety of autoimmune diseases (92), which may lead to autoimmune adverse events after PD-1/PD-L1 inhibitor treatment. The pathological presence of Th17 lymphocytes has also been described in the TME of many cancers, including lung cancer (93). In lung cancer mouse models, Th17 and IL-17 have been proven to be involved in tumorigenesis through their proinflammatory effects, as well as the occurrence of toxic effects such as interstitial pneumonia (94).

### 3.2 Increased Preexisting and Emerging Autoantibodies

An increasing series of studies have shown that the occurrence of irAEs may be associated with increased preexisting and emerging autoantibodies in human immunity. PD-1-targeted therapy leads to the dysfunction of Tregs and mediates the production of pathological autoantibodies in both PD-1-knockout mice and patients (95, 96). A multivariate analysis indicated that the presence of preexisting antibodies, such as rheumatoid factor (RF), antinuclear antibody, antithyroglobulin, and antithyroid peroxidase, was independently associated with the development of irAEs in different organs. It can be concluded that increased preexisting and emerging autoantibodies are probably involved in the mechanism of CIP. Salahaldin A. Tahir et al. found a median 1.34-fold significant increase in autoantibodies against CD74 after immune checkpoint therapy in patients with immune-related pneumonia, which suggested that CD74 autoantibodies play a role in pneumonitis (55). CD74, an autoantibody active protein, can stimulate the release of inflammatory mediators (97) as an intracellular chaperone of major histocompatibility complex class II (MHC-II) but is expressed on the cell membrane of immune cells, including macrophages (55). Taken together, the above results suggest that elevated levels of preexisting or emerging autoantibodies play a role in the development of immune-related adverse events.

### 3.3 Unbalanced Inflammatory Cytokines

Cytokines, a class of small molecular proteins with a wide range of biological activities, are mainly synthesized and secreted by immune cells. Cytokines have multiple biological functions, such as regulating innate immunity and adaptive immunity, influencing tumor growth and participating in inflammation (98). The pathophysiological mechanisms of immune-related adverse events are also thought to be mediated *via* cytokines. Lim and colleagues analyzed the expression of 65 cytokines in longitudinal plasma samples collected prior to therapy and during treatment in melanoma patients treated with ICIs alone or in combination. Eleven circulating cytokines (G-CSF, GM-CSF, Fractalkine, FGF-2, IFNα2, IL-12p70, IL-1α, IL1, IL-1RA, IL-2 and IL-13) were significantly upregulated at baseline and early during treatment and were associated with the development of high-grade irAEs (99). In a similar study, 40 cytokines were assessed in plasma. Shaheen Khan et al. found that the upregulation of various cytokines, especially induced C-X-C motif chemokine ligand (CXCL) 9, 10, 11 and 13 after ICI treatment, was closely associated with the development of irAEs (100). CXCL9, CXCL10 and CXCL11 can activate T cells by binding to C-X-C motif chemokine receptor (CXCR) 3 and have been implicated in a variety of autoimmune diseases, including thyroiditis, systemic sclerosis, and inflammatory bowel disease (IBD) (101). ICIs can activate T cells, leading to excessive release of cytokines and powerful proinflammatory reactions, which promote the development of irAEs.

Although a large number of cytokines related to the occurrence of irAEs have been found, a considerable lack of data has been reported about CIP in NSCLC patients during anti-PD-1/PD-L1 treatment. At present, there are three main types of cytokines related to the occurrence of CIP: C-reactive protein (CRP), IL-6 and IL-17.

#### 3.3.1 CRP and IL-6

CRP is an acute phase protein that is secreted by liver cells in response to inflammatory cytokines such as IL-6 and tumor necrosis factor-α (TNF-α) (102). A retrospective study reported that serum CRP levels were significantly elevated in patients with irAEs, such as pituitary inflammation, hepatitis, thyroiditis and autoimmune colitis (103). IL-6 has a proinflammatory effect in the TME (104) and plays an active role in innate and adaptive immunity, such as the activation of Th cells, inhibition of Tregs, and differentiation of B cells (105). Several studies have shown that elevated levels of IL-6 after ICI treatment are closely related to irAEs, such as psoriasiform (89) and Crohn’s disease (106). Wussler, Kozhuharov et al. (107) have shown that IL-6 and CRP were significantly higher in patients with pneumonia. CRP and IL-6 levels are elevated in NSCLC patients who developed CIP after atezolizumab treatment compared with baseline levels (108). Therefore, the development of CIP may be attributed to the excessive activation of the immune system induced by CRP and IL-6 as well as its powerful pro-inflammatory properties. Taken together, the above results suggest that dysregulated immune activation and supraphysiological levels of CRP and IL-6 are involved in the mechanism of CIP in NSCLC patients.

#### 3.3.2 IL-17

IL-17 is an important cytokine with diverse functions, playing an important role in autoimmune diseases and inflammation. IL-17 is expressed in CD8^+^ T cells, natural immune γδ T cells, NK cells and ILC3s in the lungs. The abnormal expression of IL-17 is associated with the pathology of many lung diseases, including asthma, pneumonia and pulmonary fibrosis (109). In addition, IL-17 has been found to be associated with the development of some irAEs, such as colitis (110) and psoriasiform dermatologic toxicity (111). Lou et al. showed that the levels of IL-17 in serum significantly increased in NSCLC patients with CIP following ICI treatment (112). The underlying mechanism may be that blocking PD-1 and PD-L1 could destroy immune tolerance and increase the activation of T cells (113), including the increase in Th17 cells in peripheral blood (114). An increase in the percentages of Th1 and Th17 cells can induce higher levels of IL-17A in plasma and BALF (71). It is worth noting that the levels of IL-17A and IL-35 increased when CIP was diagnosed and decreased during clinical recovery, indicating that IL-17A is related to the occurrence and development of CIP. Therefore, IL-17 may mediate off-target lung destruction in CIP.

### 3.4 Different Treatment Modes

**Figure 3:**
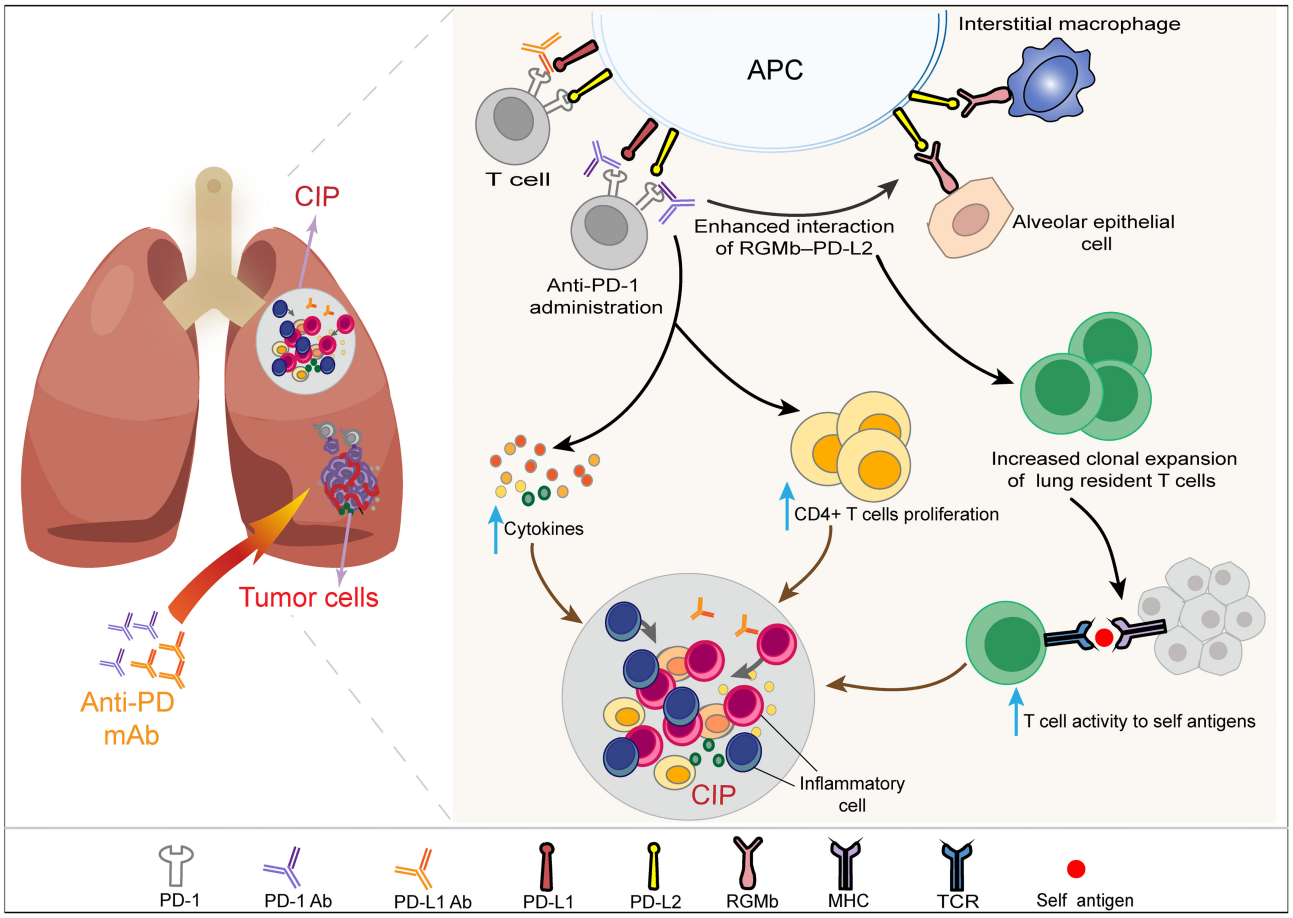
Schematic diagram of the difference in the mechanism of CIP mediated by PD-L1 inhibitor and PD-1 inhibitor monotherapy. The mechanisms of checkpoint inhibitor pneumonitis (CIP) induced by PD-1 inhibitors and PD-L1 inhibitors in patients with NSCLC are not completely the same and may be related to PD-1 ligands, including PD-L1 and PD-L2. PD-L1 blockade does not influence the binding between PD-L2 and its receptor PD-1, which is associated with immune tolerance in lung tissue. PD-1 inhibitors can simultaneously block the interaction between PD-1 and its ligands, including PD-L1 and PD-L2. The blockade of PD-1-PD-L2 signaling by PD-1 inhibitors has been observed to increase cytokine production and/or CD4+ T cell proliferation, which can increase the incidence and severity of CIP compared with PD-L1 inhibitors. Moreover, the application of a PD-1 inhibitor can increase the binding of PD-L2 to repulsive guidance molecule b (RGMb). RGMb has been observed to be expressed in lung interstitial macrophages, alveolar epithelial cells and other cells of the immune system. The interaction of RGMb–PD-L2 can increase T cell activity to self-antigens by increasing the clonal expansion of T cells that reside in the lung and then damage normal lung tissue. NSCLC, non-small cell cancer; CIP, checkpoint inhibitor pneumonitis; PD-1, programmed cell death protein 1; PD-L1, programmed cell death protein ligand-1; PD-L2, programmed cell death protein ligand-2; RGMb, repulsive guidance molecule b.

A higher incidence and severity of CIP have been observed with anti-PD-1 therapy than PD-L1 inhibitors in patients with NSCLC, suggesting that the mechanisms of CIP induced by the two agents are not completely the same (28). The incidence and severity difference of CIP may be related to PD-1 ligands, including PD-L1 and PD-L2. It has been confirmed in animal models that the expression of programmed death ligand 2 (PD-L2) in tumor cells may inhibit antitumor immunity and may be involved in the resistance to monoanti-PD-L1 therapy (115). It is conceivable that the blockade of PD-L1 and PD-L2 with PD-1 inhibitors can reverse such resistance and enhance antitumor immunity. However, at the same time, the blockade of PD-1 triggers stronger T cell expansion and cytokine production without timely braking or clearing, which causes the occurrence of CIP (116). Cells expressing PD-L2 mainly include activated T cells, DCs, macrophages and Th2 cells (117). PD-L1 inhibitors do not influence the interaction between PD-L2 and its receptor PD-1, which is associated with immune tolerance in lung tissue. However, PD-1 inhibitors can not only block the interaction between PD-1 and its ligands PD-L1 and PD-L2 but also increase the binding of PD-L2 to repulsive guidance molecule b (RGMb). RGMb has been observed to be expressed in lung interstitial macrophages, alveolar epithelial cells and other cells of the immune system, such as CD4+ and CD8+ T cells (118). Blockade of PD-1-PD-L2 signaling has been observed to increase cytokine production and/or CD4+ T cell proliferation (119). Moreover, the increased interaction of RGMb–PD-L2 mediated by PD-1 inhibitors might disrupt the immune tolerance of lung tissue by increasing the vigorous clonal expansion of T cells that reside in the lung and then lead to the occurrence of pneumonitis (118) (**Figure 3**). Recently, the combination of radiotherapy and immunotherapy has attracted increasing attention. This combination therapy model has shown improved efficacy in patients with NSCLC (120). However, a history of previous radiotherapy may increase the incidence of CIP (42, 121). Myers and Lu found significantly elevated T cells in pulmonary tissues of C57BL/6 mice after combination therapy with anti-PD-1 and thoracic irradiation. They suggested that healthy tissue damaged by thoracic irradiation is more easily destroyed by T cells activated during ICI treatment, which may be the underlying mechanism of the increased incidence of CIP after radiotherapy (122). Furthermore, oxidative damage of DNA, overinfiltration of immune cells, upregulation of proinflammatory factors, and deposition of collagen induced by radiotherapy may alternate the immune microenvironment of the lung and make it more vulnerable to the stronger immune response caused by PD-1/L1 inhibitors (116, 123). A higher incidence of pneumonitis has been observed during the combination therapy of ICIs with other antitumor drugs, such as chemotherapeutic drugs (10) and EGFR tyrosine kinase inhibitors (TKIs) (124). The mechanisms underlying the increased risk of pneumonitis mediated by combination therapies are not clear. One of the reasons may be due to the additive effects of toxicities, and further preclinical studies are still needed to explore the exact mechanism.

### 3.5 Other Potential Mechanisms

The regulation of gut microbiomes on immunity may be related to the effect and toxicity of immunotherapy (125–127). Gut microbiome transplantation has been reported to treat CTLA-4-associated immune colitis (128). Hakozaki et al. observed differences in gut microbiomes in advanced NSCLC patients with low- and high-grade irAEs (129). The role of microbiomes in the “gut-lung axis” formed by releasing immune substances or migrating to interact with microbiota colonized in the respiratory system has also attracted increasing attention. Increasing attention has been given to the role of the “gut-lung axis” formed by the interaction between microbiomes colonized in the gut and respiratory system (130, 131). Further research is needed to determine whether microbiomes play a role in CIP. Compared with gut microbiomes, there are few studies on the relationship between respiratory microbiomes and immunotherapy in cancer patients. Studies have shown that anti-PD-1 treatment can increase the alpha diversity and abundance of specific microbes (132, 133). Zhang et al. (134) found that featured respiratory microbes such as enriched Streptococcus may enhance antitumor immune responses by increasing antigen presentation and effector T cell function, which may increase the incidence of irAEs, including CIP.

Noncoding RNAs may also be involved in the regulation of irAEs. Marschner and colleagues observed a higher incidence of severe irAEs in different organs, including the lung, in mice lacking microRNA-146a (miR-146a). They recently detected SNPs in the miR-146a gene in humans and found that reduced miR-146a expression caused by SNP rs2910164 was related to an increased risk of severe irAEs, reduced progression-free survival and increased neutrophil counts both at baseline and during ICI treatment (135). Further research is still needed to determine whether and how miR-146a affects the development of irAEs, especially CIP.

In addition, B cells, natural killer (NK) cells, and dendritic cells may also be involved in the development of CIP after PD-1 blockade (136–138). The expression of PD-1 on NK cells has been observed in cancer patients (138). Hsu et al. (139) found that NK cells expressing PD-1 may mediate immunosuppression in tumors by interacting with PD-L1^+^ tumor cells. PD-1/PD-L1 blockade can activate the cytotoxicity of NK cells to tumors by releasing PD-1–imposed immune inhibition. When ICI treatment induces inflammation in nonmalignant lung tissue through various non-NK cell-mediated mechanisms, activated NK cells can directly kill infected cells and produce proinflammatory cytokines. Thus, NK cell activation mediated by PD-1/PD-L1 blockade may promote inflammation and aggravate damage to normal lung tissues.

## 4 Management of CIP

The treatment of CIP varies among the clinical grades of CIP. Current treatment options mainly include interruption of ICI therapy, glucocorticoid administration, and immunosuppressive medications such as intravenous immunoglobulins (IVIG), infliximab, cyclophosphamide, and mycophenolate mofetil (7, 14, 14, 139–142). There are several published guidelines that are involved in the management of CIP (14, 140, 143). These guidelines have issued different recommendations for patients with CIP according to their toxicity severity from grade 1 (mildest) to grade 5 (death associated with toxicity). For grade 1 CIP (asymptomatic), ICI might be considered holding while closely monitoring the symptoms of patients every 2–3 days with repeated chest CT scans and pulmonary function testing at least every 3 weeks. If any of the symptoms, radiographic testing or pulmonary function deteriorates, ICI therapy can be withdrawn, and systemic corticosteroid therapy should be initiated. Patients with grade 2 CIP need to withhold ICI therapy immediately and recommend systemic methylprednisolone treatment (0.5–1.0 mg/kg daily). Steroids can be administered orally or intravenously and taper over 4–6 weeks if symptoms improve after 48–72 h. Empiric treatment with antibiotics should start if infection is suspected. When no clinical improvement can be observed 48–72 hours after the beginning of steroid treatment, patients with CIP should be treated as grade 3–4. For grade 3–4 CIP, these patients should permanently discontinue ICI treatment and be hospitalized. Current guidelines recommend that methylprednisolone should be administered orally or intravenously at a dose of 2–4 mg/kg/day, along with empirical antibiotic therapy. If symptoms improve after 48–72 h, steroids should be tapered over 8 weeks. In contrast, if symptoms do not improve or deteriorate, additional immunosuppressive therapy should be considered. Currently, immunosuppressive agents mainly include IVIG, infliximab, cyclophosphamide, and mycophenolate mofetil (MMF) (14, 140, 143). In addition, IL-6 blockade (tocilizumab) may be a potential second-line treatment for CIP without affecting the efficacy of immunotherapy (144, 145). However, the agent still lacks clear evidence of efficacy and safety, which needs further investigation.

## 5 Potential Predictive Biomarkers of CIP

CIP is a life-threatening adverse event in some instances and possesses a higher rate in NSCLC than other tumor types. Thus, potential predictive biomarkers for CIP are necessary. The potential biomarkers reported to date mainly involve cell-based biomarkers, chemokines/cytokines, autoantibodies and genetics. In addition, there are some other emerging novel biomarkers on the rise, such as tumor genomics, microbiome and radiographic features (146). Recently, Lin et al. (147) conducted a real-world retrospective study to explore blood biomarkers related to the occurrence and prognosis of CIP in lung cancer patients. They found that increased levels of IL-6 and IL-10, neutrophil to lymphocyte ratio (NLR), platelet-to-lymphocyte ratio (PLR), and lactate dehydrogenase (LDH) or reduced absolute lymphocyte count (ALC) and albumin (ALB) levels during ICI treatment may act as biomarkers for the early diagnosis of CIP. Furthermore, squamous carcinoma may be related to an increased risk of CIP, and an increase in IL-6 levels along with a reduction in ALB levels at the onset of CIP were predictive of severe grade and poor prognosis of CIP. A case report has shown increased levels of several cytokines, including IL-1 receptor antagonist (IL-1 RA), IL2 RA and CXCL2, during radiation followed by ICI therapy, which indicates that these cytokines may be promising biomarkers for predicting the development of pneumonitis induced by combined therapy with radiation and ICIs (148). An increased level of anti-CD74 autoantibody pre- and posttreatment is considered a potential predictive biomarker for the development and timely treatment of CIP (55). Moreover, a high baseline absolute eosinophil count (≥0.125 ×10^9^ cells/L) can serve as a biomarker for predicting an increased risk of CIP (54). Hoefsmit et al. extensively explored the susceptible genetic loci likely to be related to irAEs. They found that several immune-related genes, including surfactant protein C (SP-C), autoimmune regulator (AIRE), telomerase reverse transcriptase (TERT) and mucin 5B oligomeric mucus/gel forming (MUC5B), might contribute to predicting the development of CIP (149).

## 6 Discussion

CIP is rare but can be life threatening and accounts for 35% of PD-1 and PD-L1 inhibitor-related deaths. The incidence and onset time of CIP can vary due to different PD-1/PD-L1 agents. The excessive immune response caused by the extra blockade of PD-L2 with a PD-1 inhibitor may be involved in the onset of CIP. Combination therapy has a higher incidence of CIP than monotherapy, with a shorter median onset time. The possible mechanism is the overlap of pulmonary toxicity of different treatments.

In this article, we pay close attention to the clinical features of pulmonary irAEs during PD-1/PD-L1 blockade, implying that clinicians should monitor patients receiving anti-PD-1/PD-L1 therapy to optimize clinical safety and efficacy. The clinical symptoms of pulmonary toxicity, radiological examination, especially chest CT and BALF, play an important role in the diagnosis of CIP. Clinicians should be fully aware of the association between the presence of CIP and cancer outcomes in NSCLC, balancing the benefits and risks of immunotherapy and thereby identifying patients who would gain most from treatment.

There is growing evidence that several possible mechanisms are involved in the development of CIP, and it is clear that there are multiple factors at play, such as disordered T cell subsets, increased preexisting and emerging autoantibodies, unbalanced inflammatory cytokines, previous chest radiotherapy, previous lung disease and combined immunotherapy. Further prospective studies on the mechanisms of CIP are needed to provide a more comprehensive and robust management of CIP. Deep studies on the mechanism of CIP in NSCLC are still limited. Preclinical studies on the molecular and histopathology of irAEs are in progress. To better understand the mechanism, the establishment of animal models is necessary. At present, some models have been established (150, 151). However, there is still a lack of empirical evidence regarding whether the existing models can truly reproduce the complex tumor microenvironment in the human body and whether the models are repeatable. Furthermore, research on biomarkers is also underway (152–154), which will be instrumental in the prediction, diagnosis and monitoring of CIP. Further research should aim to explore a series of biomarkers that can efficiently and timely predict the development, diagnosis, treatment and prognosis of CIP to achieve early detection and diagnosis of CIP.

Immunotherapy has crossed the gap of cancer treatment. As the main immune checkpoint inhibitors for the treatment of NSCLC, the combined treatment of PD-1/PD-L1 and CTLA-4 inhibitors has a better response and long-term cancer control, but it increases the incidence of CIP (155, 156). The emergence of novel checkpoint inhibitors, such as LAG-3, TIM-3, TIGHT, VISTA inhibitors and dual immunomodulators (targeting PD-1 and LAG-3), may change the existing immunotherapy strategies (157, 158). However, when it brings possible synergy, it may also bring potential irAEs. More clinical trials on the efficacy and safety of novel immune checkpoint inhibitors and combination therapies are needed. As a new therapeutic method, chimeric antigen receptor T cell (CAR-T) immunotherapy has played an increasingly important role in cancer treatment. A recent study reported that the recruitment and infiltration of CAR-T cells in NSCLC tumors can be enhanced when combined with a PD-1 inhibitor and immunogenic chemotherapy (159). Whether the combination of immunotherapies will affect the incidence and severity of irAEs needs more clinical data. For the treatment of NSCLC, various new immunotherapy modes, such as new immune checkpoint inhibitors, CAR-T therapy, ICIs combined chemoradiotherapy and targeted therapy, still need to balance efficacy and safety to optimize the optimal treatment regimen.

Understanding the mechanism of CIP will help to better manage patients with adverse events. At present, glucocorticoids are the main treatment, but some irAEs are insensitive to glucocorticoid. Mechanism-based therapy can improve symptoms, avoid treatment interruption or dose reduction due to irAEs, and finally prolong overall survival.

## Author Contributions

JY and YW collected information and wrote the manuscript. XY reviewed clinical studies and helped process the tables. LG provided language editing. JX conceived the idea for this review article. All authors contributed to the article and approved the submitted version. 

## Funding

This work was supported by National Natural Science Foundation of China (No. 81872478) and the Outstanding Youth Talent Foundation for Science and Technology of Sichuan Province (2022JDJQ0056).

## Conflict of Interest

The authors declare that the research was conducted in the absence of any commercial or financial relationships that could be construed as a potential conflict of interest.

## Publisher’s Note

All claims expressed in this article are solely those of the authors and do not necessarily represent those of their affiliated organizations, or those of the publisher, the editors and the reviewers. Any product that may be evaluated in this article, or claim that may be made by its manufacturer, is not guaranteed or endorsed by the publisher.

## References

[1] Siegel RL Miller KD Fuchs HE Jemal A. Cancer Statistics, 2021. CA Cancer J Clin (2021) 71:7–33. doi: 10.3322/caac.21654 33433946

[2] Toi Y Sugawara S Kawashima Y Aiba T Kawana S Saito R. Association of Immune-Related Adverse Events With Clinical Benefit in Patients With Advanced Non-Small-Cell Lung Cancer Treated With Nivolumab. Oncologist (2018) 23:1358–65. doi: 10.1634/theoncologist.2017-0384 PMC6291330 29934411

[3] Naidoo J Wang X Woo KM Iyriboz T Halpenny D Cunningham J. Pneumonitis in Patients Treated With Anti-Programmed Death-1/Programmed Death Ligand 1 Therapy. J Clin Oncol (2017) 35:709–17. doi: 10.1200/jco.2016.68.2005 PMC5559901 27646942

[4] Rittmeyer A Barlesi F Waterkamp D. Atezolizumab Versus Docetaxel in Patients With Previously Treated non-Small-Cell Lung Cancer (OAK): A Phase 3, Open-Label, Multicentre Randomised Controlled Trial. Lancet (2017) 389:255–65. doi: 10.1016/S0140-6736(16)32517-X PMC6886121 27979383

[5] Antonia SJ Villegas A Daniel D Vicente D Murakami S Hui R. Durvalumab After Chemoradiotherapy in Stage III Non–Small-Cell Lung Cancer. N Engl J Med (2017) 377:1919–29. doi: 10.1056/NEJMoa1709937 28885881

[6] FDA Approves Cemiplimab-rwlc for Non-small Cell Lung Cancer With High PD-L1 Expression (2021). Available at: https://www.fda.gov/drugs/resources-information-approved-drugs/fda-approves-cemiplimab-rwlc-non-small-cell-lung-cancer-high-pd-l1-expression.

[7] Friedman CF Proverbs-Singh TA Postow MA. Treatment of the Immune-Related Adverse Effects of Immune Checkpoint Inhibitors: A Review. JAMA Oncol (2016) 2:1346–53. doi: 10.1001/jamaoncol.2016.1051 27367787

[8] National Cancer Institute. Common Terminology Criteria for Adverse Events (CTCAE). Version 4.0.(2010). Available at: http://ctep.cancer.gov/protocolDevelopment/electronic_applications/ctc.htm [Accessed November 25, 2021].).

[9] Wang DY Salem J-E Cohen JV Chandra S Menzer C Ye F. Fatal Toxic Effects Associated With Immune Checkpoint Inhibitors A Systematic Review and Meta-Analysis. JAMA Oncol (2018) 4:1721–28. doi: 10.1001/jamaoncol.2018.3923 PMC6440712 30242316

[10] Paz-Ares L Luft A Vicente D Tafreshi A Gümüş M Mazières J. Pembrolizumab Plus Chemotherapy for Squamous Non-Small-Cell Lung Cancer. N Engl J Med (2018) 379:2040–51. doi: 10.1056/NEJMoa1810865 30280635

[11] Ferrara R Mezquita L Texier M Lahmar J Audigier-Valette C Tessonnier L. Hyperprogressive Disease in Patients With Advanced Non-Small Cell Lung Cancer Treated With PD-1/PD-L1 Inhibitors or With Single-Agent Chemotherapy. JAMA Oncol (2018) 4:1543–52. doi: 10.1001/jamaoncol.2018.3676 PMC6248085 30193240

[12] Zhai X Zhang J Tian Y Li J Jing W Guo H. The Mechanism and Risk Factors for Immune Checkpoint Inhibitor Pneumonitis in non-Small Cell Lung Cancer Patients. Cancer Biol Med (2020) 17:599–611. doi: 10.20892/j.issn.2095-3941.2020.0102 32944393 PMC7476083

[13] Tong ZQ Wu DY Liu D Dong M. Incidence Risk of PD-1/PD-L1-Related Pneumonia and Diarrhea in Non-Small Cell Lung Cancer (NSCLC) Patients: A Systematic Review and Meta-Analysis of Randomized Controlled Trials. Eur J Clin Pharmacol (2021) 77:1079–88. doi: 10.1007/s00228-020-03083-9 33564898

[14] Haanen J Carbonnel F Robert C Kerr KM Peters S Larkin J. Management of Toxicities From Immunotherapy: ESMO Clinical Practice Guidelines for Diagnosis, Treatment and Follow-Up. Ann Oncol (2017) 28:iv119–42. doi: 10.1093/annonc/mdx225 28881921

[15] Brahmer J Reckamp KL Baas P Crino L Eberhardt WEE Poddubskaya E. Nivolumab Versus Docetaxel in Advanced Squamous-Cell Non-Small-Cell Lung Cancer. N Engl J Med (2015) 373:123–35. doi: 10.1056/NEJMoa1504627 PMC4681400 26028407

[16] Borghaei H Paz-Ares L Horn L Spigel DR Steins M Ready NE. Nivolumab Versus Docetaxel in Advanced Nonsquamous Non-Small-Cell Lung Cancer. N Engl J Med (2015) 373:1627–39. doi: 10.1056/NEJMoa1507643 PMC5705936 26412456

[17] Hellmann MD Ciuleanu TE Pluzanski A Lee JS Otterson GA Audigier-Valette C. Nivolumab Plus Ipilimumab in Lung Cancer With a High Tumor Mutational Burden. N Engl J Med (2018) 378:2093–104. doi: 10.1056/NEJMoa1801946 PMC7193684 29658845

[18] Ahn MJ Gandhi L Hamid O Hellmann MD Garon EB Ramalingam SS. 459p Risk of Pneumonitis in Patients With Advanced NSCLC Treated With Pembrolizumab in KEYNOTE-001. Ann Oncol (2015) 26:ix125–47. doi: 10.1093/annonc/mdv532.43

[19] Herbst RS Baas P Kim DW Felip E Pérez-Gracia JL Han JY. Pembrolizumab Versus Docetaxel for Previously Treated, PD-L1-Positive, Advanced Non-Small-Cell Lung Cancer (KEYNOTE-010): A Randomised Controlled Trial. Lancet (2016) 387:1540–50. doi: 10.1016/s0140-6736(15)01281-7 26712084

[20] Reck M Rodríguez-Abreu D Robinson AG Hui R Csőszi T Fülöp A. Pembrolizumab Versus Chemotherapy for PD-L1-Positive Non-Small-Cell Lung Cancer. N Engl J Med (2016) 375:1823–33. doi: 10.1056/NEJMoa1606774 27718847

[21] Mok TSK Wu YL Kudaba I Kowalski DM Cho BC Turna HZ. Pembrolizumab Versus Chemotherapy for Previously Untreated, PD-L1-Expressing, Locally Advanced or Metastatic non-Small-Cell Lung Cancer (KEYNOTE-042): A Randomised, Open-Label, Controlled, Phase 3 Trial. Lancet (2019) 393:1819–30. doi: 10.1016/s0140-6736(18)32409-7 30955977

[22] Sezer A Kilickap S Gümüş M Bondarenko I Özgüroğlu M Gogishvili M. Cemiplimab Monotherapy for First-Line Treatment of Advanced non-Small-Cell Lung Cancer With PD-L1 of at Least 50%: A Multicentre, Open-Label, Global, Phase 3, Randomised, Controlled Trial. Lancet (2021) 397:592–604. doi: 10.1016/s0140-6736(21)00228-2 33581821

[23] Herbst RS Giaccone G de Marinis F Reinmuth N Vergnenegre A Barrios CH. Atezolizumab for First-Line Treatment of PD-L1–Selected Patients With NSCLC. N Engl J Med (2020) 383:1328–39. doi: 10.1056/NEJMoa1917346 32997907

[24] Nishio M Barlesi F West H Ball S Bordoni R Cobo M. Atezolizumab Plus Chemotherapy for First-Line Treatment of Nonsquamous NSCLC: Results From the Randomized Phase 3 IMpower132 Trial. J Thorac Oncol (2021) 16:653–64. doi: 10.1016/j.jtho.2020.11.025 33333328

[25] West H McCleod M Hussein M Morabito A Rittmeyer A Conter HJ. Atezolizumab in Combination With Carboplatin Plus Nab-Paclitaxel Chemotherapy Compared With Chemotherapy Alone as First-Line Treatment for Metastatic non-Squamous non-Small-Cell Lung Cancer (IMpower130): A Multicentre, Randomised, Open-Label, Phase 3 Trial. Lancet Oncol (2019) 20:924–37. doi: 10.1016/s1470-2045(19)30167-6 31122901

[26] Antonia SJ Villegas A Daniel D Vicente D Murakami S Hui R. Durvalumab After Chemoradiotherapy in Stage III Non-Small-Cell Lung Cancer. N Engl J Med (2017) 377:1919–29. doi: 10.1056/NEJMoa1709937 28885881

[27] Rizvi NA Cho BC Reinmuth N Lee KH Luft A Ahn MJ. Durvalumab With or Without Tremelimumab vs Standard Chemotherapy in First-Line Treatment of Metastatic Non-Small Cell Lung Cancer: The MYSTIC Phase 3 Randomized Clinical Trial. JAMA Oncol (2020) 6:661–74. doi: 10.1001/jamaoncol.2020.0237 PMC7146551 32271377

[28] Khunger M Rakshit S Pasupuleti V Hernandez AV Mazzone P Stevenson J. Incidence of Pneumonitis With Use of Programmed Death 1 and Programmed Death-Ligand 1 Inhibitors in Non-Small Cell Lung Cancer: A Systematic Review and Meta-Analysis of Trials. Chest (2017) 152:271–81. doi: 10.1016/j.chest.2017.04.177 28499515

[29] Durm GA Jabbour SK Althouse SK Liu Z Sadiq AA Zon RT. A Phase 2 Trial of Consolidation Pembrolizumab Following Concurrent Chemoradiation for Patients With Unresectable Stage III Non-Small Cell Lung Cancer: Hoosier Cancer Research Network LUN 14-179. Cancer (2020) 126:4353–61. doi: 10.1002/cncr.33083 PMC10865991 32697352

[30] Peters S Felip E Dafni U Belka C Guckenberger M Irigoyen A. Safety Evaluation of Nivolumab Added Concurrently to Radiotherapy in a Standard First Line Chemo-Radiotherapy Regimen in Stage III Non-Small Cell Lung Cancer-The ETOP NICOLAS Trial. Lung Cancer (2019) 133:83–7. doi: 10.1016/j.lungcan.2019.05.001 31200833

[31] Nishino M Giobbie-Hurder A Hatabu H Ramaiya NH Hodi FS. Incidence of Programmed Cell Death 1 Inhibitor-Related Pneumonitis in Patients With Advanced Cancer: A Systematic Review and Meta-Analysis. JAMA Oncol (2016) 2:1607–16. doi: 10.1001/jamaoncol.2016.2453 27540850

[32] Yang H Jin T Li M Xue J Lu BJPCM. Synergistic Effect of Immunotherapy and Radiotherapy in Non-Small Cell Lung Cancer: Current Clinical Trials and Prospective Challenges. Precis Clin Med (2019) 2:57–70. doi: 10.1093/pcmedi/pbz004 PMC8985786 35694698

[33] Cousin F Desir C Ben Mustapha S Mievis C Coucke P Hustinx R. Incidence, Risk Factors, and CT Characteristics of Radiation Recall Pneumonitis Induced by Immune Checkpoint Inhibitor in Lung Cancer. Radiother Oncol (2021) 157:47–55. doi: 10.1016/j.radonc.2021.01.001 33453313

[34] Kato T Masuda N Nakanishi Y Takahashi M Hida T Sakai H. Nivolumab-Induced Interstitial Lung Disease Analysis of Two Phase II Studies Patients With Recurrent or Advanced Non-Small-Cell Lung Cancer. Lung Cancer (2017) 104:111–8. doi: 10.1016/j.lungcan.2016.12.016 28212992

[35] Suresh K Voong KR Shankar B Forde PM Ettinger DS Marrone KA. Pneumonitis in Non-Small Cell Lung Cancer Patients Receiving Immune Checkpoint Immunotherapy: Incidence and Risk Factors. J Thorac Oncol (2018) 13:1930–9. doi: 10.1016/j.jtho.2018.08.2035 30267842

[36] Morita R Okishio K Shimizu J Saito H Sakai H Kim YH. Real-World Effectiveness and Safety of Nivolumab in Patients With non-Small Cell Lung Cancer: A Multicenter Retrospective Observational Study in Japan. Lung Cancer (2020) 140:8–18. doi: 10.1016/j.lungcan.2019.11.014 31838169

[37] Naidoo J Cottrell TR Lipson EJ Forde PM Illei PB Yarmus LB. Chronic Immune Checkpoint Inhibitor Pneumonitis. J Immunother Cancer (2020) 8:e000840. doi: 10.1136/jitc-2020-000840 32554618 PMC7304886

[38] Suresh K Naidoo J Lin CT Danoff S. Immune Checkpoint Immunotherapy for Non-Small Cell Lung Cancer: Benefits and Pulmonary Toxicities. Chest (2018) 154:1416–23. doi: 10.1016/j.chest.2018.08.1048 PMC6335259 30189190

[39] Cho JY Kim J Lee JS Kim YJ Kim SH Lee YJ. Characteristics, Incidence, and Risk Factors of Immune Checkpoint Inhibitor-Related Pneumonitis in Patients With Non-Small Cell Lung Cancer. Lung Cancer (2018) 125:150–6. doi: 10.1016/j.lungcan.2018.09.015 30429014

[40] Voong KR Hazell S Hu C Hayman J Hales R Marrone K. MA 09.08 Receipt of Chest Radiation and Immune-Related Pneumonitis in Patients With NSCLC Treated With Anti-PD-1/PD-L1. J Thorac Oncol (2017) 12:S1837. doi: 10.1016/j.jtho.2017.09.529

[41] Torre-Bouscoulet L Arroyo-Hernandez M Martinez-Briseno D Munoz-Montano WR Gochicoa-Rangel L Bacon-Fonseca L. Longitudinal Evaluation of Lung Function in Patients With Advanced Non-Small Cell Lung Cancer Treated With Concurrent Chemoradiation Therapy. Int J Radiat Oncol Biol Phys (2018) 101:910–8. doi: 10.1016/j.ijrobp.2018.04.014 29976503

[42] Garon EB Rizvi NA Hui R Leighl N Balmanoukian AS Eder JP. Pembrolizumab for the Treatment of Non-Small-Cell Lung Cancer. N Engl J Med (2015) 372:2018–28. doi: 10.1056/NEJMoa1501824 25891174

[43] Owen DH Wei L Bertino EM Edd T Villalona-Calero MA He K. Incidence, Risk Factors, and Effect on Survival of Immune-Related Adverse Events in Patients With Non-Small-Cell Lung Cancer. Clin Lung Cancer (2018) 19:e893–900. doi: 10.1016/j.cllc.2018.08.008 PMC7193681 30197259

[44] Barrón F Sánchez R Arroyo-Hernández M Blanco C Zatarain-Barrón ZL Catalán R. Risk of Developing Checkpoint Immune Pneumonitis and Its Effect on Overall Survival in Non-Small Cell Lung Cancer Patients Previously Treated With Radiotherapy. Front Oncol (2020) 10:570233. doi: 10.3389/fonc.2020.570233 33117699 PMC7550759

[45] Cui P Liu Z Wang G Ma J Qian Y Zhang F. Risk Factors for Pneumonitis in Patients Treated With Anti-Programmed Death-1 Therapy: A Case-Control Study. Cancer Med (2018) 7:4115–20. doi: 10.1002/cam4.1579 PMC6089164 29797416

[46] Ahn MJ Yang J Yu H Saka H Ramalingam S Goto K. 136o: Osimertinib Combined With Durvalumab in EGFR-Mutant Non-Small Cell Lung Cancer: Results From the TATTON Phase Ib Trial. J Thorac Oncol (2016) 11:S115. doi: 10.1016/s1556-0864(16)30246-5

[47] Schoenfeld AJ Arbour KC Rizvi H Iqbal AN Gadgeel SM Girshman J. Severe Immune-Related Adverse Events Are Common With Sequential PD-(L)1 Blockade and Osimertinib. Ann Oncol (2019) 30:839–44. doi: 10.1093/annonc/mdz077 PMC7360149 30847464

[48] Lin X Lu T Li S Xie X Chen X Jiang J. Cytomegalovirus Infection as an Underestimated Trigger for Checkpoint Inhibitor-Related Pneumonitis in Lung Cancer: A Retrospective Study. Clin Transl Oncol (2021) 23:389–96. doi: 10.1007/s12094-020-02432-5 32613413

[49] Shibata Y Murakami S Kato T. Overview of Checkpoint Inhibitor Pneumonitis: Incidence and Associated Risk Factors. Expert Opin Drug Saf (2021) 20:537–47. doi: 10.1080/14740338.2021.1898584 33650443

[50] Sul J Blumenthal GM Jiang X He K Keegan P Pazdur R. FDA Approval Summary: Pembrolizumab for the Treatment of Patients With Metastatic Non-Small Cell Lung Cancer Whose Tumors Express Programmed Death-Ligand 1. Oncologist (2016) 21:643–50. doi: 10.1634/theoncologist PMC4861368 27026676

[51] Kanai O Kim YH Demura Y Kanai M Ito T Fujita K. Efficacy and Safety of Nivolumab in Non-Small Cell Lung Cancer With Preexisting Interstitial Lung Disease. Thorac Cancer (2018) 9:847–55. doi: 10.1111/1759-7714.12759 PMC6026605 29782069

[52] Yamaguchi T Shimizu J Hasegawa T Horio Y Inaba Y Yatabe Y. Pre-Existing Pulmonary Fibrosis Is a Risk Factor for Anti-PD-1-Related Pneumonitis in Patients With Non-Small Cell Lung Cancer: A Retrospective Analysis. Lung Cancer (2018) 125:212–7. doi: 10.1016/j.lungcan.2018.10.001 30429022

[53] Moda M Saito H Kato T Usui R Kondo T Nakahara Y. Tumor Invasion in the Central Airway Is a Risk Factor for Early-Onset Checkpoint Inhibitor Pneumonitis in Patients With Non-Small Cell Lung Cancer. Thorac Cancer (2020) 11:3576–84. doi: 10.1111/1759-7714.13703 PMC7705619 33078531

[54] Chu X Zhao J Zhou J Zhou F Jiang T Jiang S. Association of Baseline Peripheral-Blood Eosinophil Count With Immune Checkpoint Inhibitor-Related Pneumonitis and Clinical Outcomes in Patients With Non-Small Cell Lung Cancer Receiving Immune Checkpoint Inhibitors. Lung Cancer (2020) 150:76–82. doi: 10.1016/j.lungcan.2020.08.015 33080551

[55] Tahir SA Gao J Miura Y Blando J Tidwell RSS Zhao H. Autoimmune Antibodies Correlate With Immune Checkpoint Therapy-Induced Toxicities. Proc Natl Acad Sci USA (2019) 116:22246–51. doi: 10.1073/pnas.1908079116 PMC6825284 31611368

[56] Freeman-Keller M Kim Y Cronin H Richards A Gibney G Weber JS. Nivolumab in Resected and Unresectable Metastatic Melanoma: Characteristics of Immune-Related Adverse Events and Association With Outcomes. Clin Cancer Res (2016) 22:886–94. doi: 10.1158/1078-0432.Ccr-15-1136 PMC4755809 26446948

[57] Hua C Boussemart L Mateus C Routier E Boutros C Cazenave H. Association of Vitiligo With Tumor Response in Patients With Metastatic Melanoma Treated With Pembrolizumab. JAMA Dermatol (2016) 152:45–51. doi: 10.1001/jamadermatol.2015.2707 26501224

[58] Nakamura Y Tanaka R Asami Y Teramoto Y Imamura T Sato S. Correlation Between Vitiligo Occurrence and Clinical Benefit in Advanced Melanoma Patients Treated With Nivolumab: A Multi-Institutional Retrospective Study. J Dermatol (2017) 44:117–22. doi: 10.1111/1346-8138.13520 27510892

[59] Sanlorenzo M Vujic I Daud A Algazi A Gubens M Luna SA. Pembrolizumab Cutaneous Adverse Events and Their Association With Disease Progression. JAMA Dermatol (2015) 151:1206–12. doi: 10.1001/jamadermatol.2015.1916 PMC5061067 26222619

[60] Teulings H-E Limpens J Jansen SN Zwinderman AH Reitsma JB Spuls PI. Vitiligo-Like Depigmentation in Patients With Stage III-IV Melanoma Receiving Immunotherapy and Its Association With Survival: A Systematic Review and Meta-Analysis. J Clin Oncol (2015) 33:773–81. doi: 10.1200/jco.2014.57.4756 25605840

[61] Haratani K Hayashi H Chiba Y Kudo K Yonesaka K Kato R. Association of Immune-Related Adverse Events With Nivolumab Efficacy in Non-Small-Cell Lung Cancer. JAMA Oncol (2018) 4:374–8. doi: 10.1001/jamaoncol.2017.2925 PMC6583041 28975219

[62] Ricciuti B Genova C De Giglio A Bassanelli M Dal Bello MG Metro G. Impact of Immune-Related Adverse Events on Survival in Patients With Advanced Non-Small Cell Lung Cancer Treated With Nivolumab: Long-Term Outcomes From a Multi-Institutional Analysis. J Cancer Res Clin Oncol (2019) 145:479–85. doi: 10.1007/s00432-018-2805-3 PMC11810236 30506406

[63] Tone M Izumo T Awano N Kuse N Inomata M Jo T. High Mortality and Poor Treatment Efficacy of Immune Checkpoint Inhibitors in Patients With Severe Grade Checkpoint Inhibitor Pneumonitis in non-Small Cell Lung Cancer. Thorac Cancer (2019) 10:2006–12. doi: 10.1111/1759-7714.13187 PMC6775002 31482678

[64] Brahmer JR Lacchetti C Thompson JA. Management of Immune-Related Adverse Events in Patients Treated With Immune Checkpoint Inhibitor Therapy: American Society of Clinical Oncology Clinical Practice Guideline Summary. J Oncol Pract (2018) 14:247–9. doi: 10.1200/JOP.18.00005 29517954

[65] Nobashi TW Nishimoto Y Kawata Y Yutani H Nakamura M Tsuji Y. Clinical and Radiological Features of Immune Checkpoint Inhibitor-Related Pneumonitis in Lung Cancer and Non-Lung Cancers. Br J Radiol (2020) 93:20200409. doi: 10.1259/bjr.20200409 32783627 PMC8519648

[66] Nishino M Ramaiya NH Awad MM Sholl LM Maattala JA Taibi M. PD-1 Inhibitor-Related Pneumonitis in Advanced Cancer Patients: Radiographic Patterns and Clinical Course. Clin Cancer Res (2016) 22:6051–60. doi: 10.1158/1078-0432.ccr-16-1320 PMC5161686 27535979

[67] Watanabe S Ota T Hayashi M Ishikawa H Otsubo A Shoji S. Prognostic Significance of the Radiologic Features of Pneumonitis Induced by Anti-PD-1 Therapy. Cancer Med (2020) 9:3070–7. doi: 10.1002/cam4.2974 PMC7196069 32150668

[68] Marvisi M Ramponi S Balzarini L Mancini C. A "Crazy Paving" Pattern on CT Scan in a Patient Treated With Pembrolizumab. Curr Drug Saf (2019) 14:242–5. doi: 10.2174/1574886314666190312115648 PMC6876257 30864509

[69] Bronstein Y Ng CS Hwu P Hwu W-J. Radiologic Manifestations of Immune-Related Adverse Events in Patients With Metastatic Melanoma Undergoing Anti-CTLA-4 Antibody Therapy. AJR Am J Roentgenol (2011) 197:W992–1000. doi: 10.2214/ajr.10.6198 22109345

[70] Raad RA Kannan R Madden K Pavlick A. Ipilimumab-Induced Organizing Pneumonia on 18F-FDG PET/CT in a Patient With Malignant Melanoma. Clin Nucl Med (2017) 42:e345–6. doi: 10.1097/RLU.0000000000001673 28481788

[71] Wang YN Lou DF Li DY Jiang W Dong JY Gao W. Elevated Levels of IL-17A and IL-35 in Plasma and Bronchoalveolar Lavage Fluid Are Associated With Checkpoint Inhibitor Pneumonitis in Patients With Non-Small Cell Lung Cancer. Oncol Lett (2020) 20:611–22. doi: 10.3892/ol.2020.11618 PMC7285943 32565986

[72] Ivanova EA. Orekhov AN. T Helper Lymphocyte Subsets and Plasticity in Autoimmunity and Cancer: An Overview. BioMed Res Int (2015) 2015:327470. doi: 10.1155/2015/327470 26583100 PMC4637008

[73] Suresh K Naidoo J Zhong Q Xiong Y Mammen J de Flores MV. The Alveolar Immune Cell Landscape Is Dysregulated in Checkpoint Inhibitor Pneumonitis. J Clin Invest (2019) 129:4305–15. doi: 10.1172/JCI128654 PMC6763233 31310589

[74] Suzuki K Yanagihara T Matsumoto K Kusaba H Yamauchi T Ikematsu Y. Immune-Checkpoint Profiles for T Cells in Bronchoalveolar Lavage Fluid of Patients With Immune-Checkpoint Inhibitor-Related Interstitial Lung Disease. Int Immunol (2020) 32:547–57. doi: 10.1093/intimm/dxaa022 32253426

[75] Kharkevitch DD Dai S Balch GC Maeda T Balch CM Itoh K. Characterization of Autologous Tumor-Specific T-Helper 2 Cells in Tumor-Infiltrating Lymphocytes From a Patient With Metastatic Melanoma. Int J Cancer (1994) 58:317–23. doi: 10.1002/ijc.2910580302 7914181

[76] Najafi M Farhood B Mortezaee K. Contribution of Regulatory T Cells to Cancer: A Review. J Cell Physiol (2019) 234:7983–93. doi: 10.1002/jcp.27553 30317612

[77] Dulos J Carven GJ van Boxtel SJ Evers S Driessen-Engels LJA Hobo W. PD-1 Blockade Augments Th1 and Th17 and Suppresses Th2 Responses in Peripheral Blood From Patients With Prostate and Advanced Melanoma Cancer. J Immunother (2012) 35:169–78. doi: 10.1097/CJI.0b013e318247a4e7 22306905

[78] Yoshino K Nakayama T Ito A Sato E Kitano S. Severe Colitis After PD-1 Blockade With Nivolumab in Advanced Melanoma Patients: Potential Role of Th1-Dominant Immune Response in Immune-Related Adverse Events: Two Case Reports. BMC Cancer (2019) 19:1019. doi: 10.1186/s12885-019-6138-7 31664934 PMC6819390

[79] Kim ST Sheshadri A Shannon V Kontoyiannis DP Kantarjian H Garcia-Manero G. Distinct Immunophenotypes of T Cells in Bronchoalveolar Lavage Fluid From Leukemia Patients With Immune Checkpoint Inhibitors-Related Pulmonary Complications. Front Immunol (2021) 11:590494. doi: 10.3389/fimmu.2020.590494 33552049 PMC7859512

[80] Luger D Silver PB Tang J Cua D Chen Z Iwakura Y. Either a Th17 or a Th1 Effector Response can Drive Autoimmunity: Conditions of Disease Induction Affect Dominant Effector Category. J Exp Med (2008) 205:799–810. doi: 10.1084/jem.20071258 18391061 PMC2292220

[81] Nedoszytko B Lange M Sokołowska-Wojdyło M Renke J Trzonkowski P Sobjanek M. The Role of Regulatory T Cells and Genes Involved in Their Differentiation in Pathogenesis of Selected Inflammatory and Neoplastic Skin Diseases. Part I: Treg Properties and Functions. Postepy Dermatol Alergol (2017) 34:285–94. doi: 10.5114/ada.2017.69305 PMC5560174 28951701

[82] Francisco LM Salinas VH Brown KE Vanguri VK Freeman GJ Kuchroo VK. PD-L1 Regulates the Development, Maintenance, and Function of Induced Regulatory T Cells. J Exp Med (2009) 206:3015–29. doi: 10.1084/jem.20090847 PMC2806460 20008522

[83] Amarnath S Mangus CW Wang JC Wei F He A Kapoor V. The PDL1-PD1 Axis Converts Human TH1 Cells Into Regulatory T Cells. Sci Transl Med (2011) 3:111–20. doi: 10.1126/scitranslmed.3003130 PMC3235958 22133721

[84] Zhang L Zhang M Xu J Li S Gu M. The Role of the Programmed Cell Death Protein-1/Programmed Death-Ligand 1 Pathway, Regulatory T Cells and T Helper 17 Cells in Tumor Immunity: A Narrative Review. Ann Transl Med (2020) 8:1526. doi: 10.21037/atm-20-6719 33313271 PMC7729304

[85] Passat T Touchefeu Y Gervois N Jarry A Bossard C Bennouna J. Physiopathological Mechanisms of Immune-Related Adverse Events Induced by Anti-CTLA-4, Anti-PD-1 and Anti-PD-L1 Antibodies in Cancer Treatment. Bull Cancer (2018) 105:1033–41. doi: 10.1016/j.bulcan.2018.07.005 30244981

[86] Martin-Orozco N Muranski P Chung Y Yang XO Yamazaki T Lu S. T Helper 17 Cells Promote Cytotoxic T Cell Activation in Tumor Immunity. Immunity (2009) 31:787–98. doi: 10.1016/j.immuni.2009.09.014 PMC2787786 19879162

[87] Munegowda MA Deng Y Mulligan SJ Xiang J. Th17 and Th17-Stimulated CD8 T Cells Play a Distinct Role in Th17-Induced Preventive and Therapeutic Antitumor Immunity. Cancer Immunol Immunother (2011) 60:1473–84. doi: 10.1007/s00262-011-1054-y PMC11028972 21660450

[88] Numasaki M Watanabe M Suzuki T Takahashi H Nakamura A Mcallister F. IL-17 Enhances the Net Angiogenic Activity and *In Vivo* Growth of Human non-Small Cell Lung Cancer in SCID Mice Through Promoting CXCR-2-Dependent Angiogenesis. J Immunol (2005) 175:6177–89. doi: 10.4049/jimmunol.175.9.6177 16237115

[89] McAlees JW Lajoie S Dienger K Sproles AA Richgels PK Yang Y. Differential Control of CD4(+) T-Cell Subsets by the PD-1/PD-L1 Axis in a Mouse Model of Allergic Asthma. Eur J Immunol (2015) 45:1019–29. doi: 10.1002/eji.201444778 PMC4440042 25630305

[90] Lee YK Turner H Maynard CL Oliver JR Chen D Elson CO. Late Developmental Plasticity in the T Helper 17 Lineage. Immunity (2009) 30:92–107. doi: 10.1016/j.immuni.2008.11.005 19119024 PMC3607320

[91] Basdeo SA Cluxton D Sulaimani J Moran B Canavan M Orr C. Ex-Th17 (Nonclassical Th1) Cells Are Functionally Distinct From Classical Th1 and Th17 Cells and Are Not Constrained by Regulatory T Cells. J Immunol (2017) 198:2249–59. doi: 10.4049/jimmunol.1600737 28167631

[92] Knochelmann HM Dwyer CJ Bailey SR Amaya SM Paulos CM. When Worlds Collide: Th17 and Treg Cells in Cancer and Autoimmunity. Cell Mol Immunol (2018) 15:458–69. doi: 10.1038/s41423-018-0004-4 PMC6068176 29563615

[93] Kryczek I Banerjee M Cheng P Vatan L Zou W. Phenotype, Distribution, Generation, and Functional and Clinical Relevance of Th17 Cells in the Human Tumor Environments. Blood (2009) 114:1141–9. doi: 10.1182/blood-2009-03-208249 PMC2723011 19470694

[94] Chang SH Mirabolfathinejad SG Katta H Cumpian AM Chen D. T Helper 17 Cells Play a Critical Pathogenic Role in Lung Cancer. Proc Natl Acad Sci USA (2014) 111:5664–9. doi: 10.1073/pnas.1319051111 PMC3992670 24706787

[95] Okazaki T Tanaka Y Nishio R Mitsuiye T Mizoguchi A Wang J. Autoantibodies Against Cardiac Troponin I are Responsible for Dilated Cardiomyopathy in PD-1-Deficient Mice. Nat Med (2003) 9:1477–83. doi: 10.1038/nm955 14595408

[96] Kong YC Flynn JC. Opportunistic Autoimmune Disorders Potentiated by Immune-Checkpoint Inhibitors Anti-CTLA-4 and Anti-PD-1. Front Immunol (2014) 5:206. doi: 10.3389/fimmu.2014.00206 24904570 PMC4032988

[97] Starlets D Gore Y Binsky I Haran M Harpaz N Shvidel L. Cell-Surface CD74 Initiates a Signaling Cascade Leading to Cell Proliferation and Survival. Blood (2006) 107:4807–16. doi: 10.1182/blood-2005-11-4334 16484589

[98] Rotondi M Chiovato L Romagnani S Serio M Romagnani P. Role of Chemokines in Endocrine Autoimmune Diseases. Endocr Rev (2007) 28:492–520. doi: 10.1210/er.2006-0044 17475924

[99] Lim SY Lee JH Gide TN Menzies AM Guminski A Carlino MS. Circulating Cytokines Predict Immune-Related Toxicity in Melanoma Patients Receiving Anti-PD-1-Based Immunotherapy. Clin Cancer Res (2019) 25:1557–63. doi: 10.1158/1078-0432.CCR-18-2795 30409824

[100] Khan S Khan SA Luo X Fattah FJ Saltarski J Gloria-McCutchen Y. Immune Dysregulation in Cancer Patients Developing Immune-Related Adverse Events. Br J Cancer (2019) 120:63–8. doi: 10.1038/s41416-018-0155-1 PMC6325132 30377338

[101] Magliozzi R Howell O Vora A Serafini B Nicholas R Puopolo M. Meningeal B-Cell Follicles in Secondary Progressive Multiple Sclerosis Associate With Early Onset of Disease and Severe Cortical Pathology. Brain (2007) 130:1089–104. doi: 10.1093/brain/awm038 17438020

[102] Pepys MB Hirschfield GM. C-Reactive Protein: A Critical Update. J Clin Invest (2003) 111:1805–12. doi: 10.1172/JCI18921 PMC161431 12813013

[103] Abolhassani AR Schuler G Kirchberger MC Heinzerling L. C-Reactive Protein as an Early Marker of Immune-Related Adverse Events. J Cancer Res Clin Oncol (2019) 145:2625–31. doi: 10.1007/s00432-019-03002-1 PMC11810282 31492984

[104] Hunter CA Jones SA. IL-6 as a Keystone Cytokine in Health and Disease. Nat Immunol (2015) 16:448–57. doi: 10.1038/ni.3153 25898198

[105] Scheller J Chalaris A Schmidt-Arras D Rose-John S. The Pro- and Anti-Inflammatory Properties of the Cytokine Interleukin-6. Biochim Biophys Acta (2011) 1813:878–88. doi: 10.1016/j.bbamcr.2011.01.034 21296109

[106] Bettelli E Carrier Y Gao W Korn T Strom T Oukka M. Reciprocal Developmental Pathways for the Generation of Pathogenic Effector T(H)17 and Regulatory T Cells. Nature (2006) 441:235–8. doi: 10.1038/nature04753 16648838

[107] Wussler D Kozhuharov N Tavares Oliveira M Bossa A Sabti Z Nowak A. Clinical Utility of Procalcitonin in the Diagnosis of Pneumonia. Clin Chem (2019) 65:1532–42. doi: 10.1373/clinchem.2019.306787 31615771

[108] Zhang JJ Dong X Cao YY Yuan YD Yang YB Yan YQ. Clinical Characteristics of 140 Patients Infected With SARS-CoV-2 in Wuhan, China. Allergy (2020) 75:1730–41. doi: 10.1111/all.14238 32077115

[109] Iwanaga N Kolls JK. Updates on T Helper Type 17 Immunity in Respiratory Disease. Immunology (2019) 156:3–8. doi: 10.1111/imm.13006 30260473 PMC6283652

[110] Tarhini AA Zahoor H Lin Y Malhotra U Sander C Butterfield LH. Baseline Circulating IL-17 Predicts Toxicity While TGF-Beta1 and IL-10 Are Prognostic of Relapse in Ipilimumab Neoadjuvant Therapy of Melanoma. J Immunother Cancer (2015) 3:39. doi: 10.1186/s40425-015-0081-1 26380086 PMC4570556

[111] Johnson D Patel AB Uemura MI Trinh VA Jackson N Zobniw CM. IL17A Blockade Successfully Treated Psoriasiform Dermatologic Toxicity From Immunotherapy. Cancer Immunol Res (2019) 7:860–5. doi: 10.1158/2326-6066.CIR-18-0682 30996018

[112] Luo W Wang Z Tian P Li W. Safety and Tolerability of PD-1/PD-L1 Inhibitors in the Treatment of Non-Small Cell Lung Cancer: A Meta-Analysis of Randomized Controlled Trials. J Cancer Res Clin Oncol (2018) 144:1851–9. doi: 10.1007/s00432-018-2707-4 PMC11813414 30019319

[113] Makkouk A Weiner GJ. Cancer Immunotherapy and Breaking Immune Tolerance: New Approaches to an Old Challenge. Cancer Res (2015) 75:5–10. doi: 10.1158/0008-5472 25524899 PMC4286422

[114] Citrin DE Mitchell JB. Mechanisms of Normal Tissue Injury From Irradiation. Semin Radiat Oncol (2017) 27:316–24. doi: 10.1016/j.semradonc.2017.04.001 PMC5653270 28865514

[115] Tanegashima T Togashi Y Azuma K Kawahara A Ideguchi K Sugiyama D. Immune Suppression by PD-L2 Against Spontaneous and Treatment-Related Antitumor Immunity. Clin Cancer Res (2019) 25:4808–19. doi: 10.1158/1078-0432.CCR-18-3991 31076547

[116] Li M Gan L Song A Xue J Lu Y. Rethinking Pulmonary Toxicity in Advanced Non-Small Cell Lung Cancer in the Era of Combining Anti-PD-1/PD-L1 Therapy With Thoracic Radiotherapy. Biochim Biophys Acta Rev Cancer (2019) 1871:323–30. doi: 10.1016/j.bbcan.2019.02.004 30826426

[117] Greenwald RJ Freeman GJ Sharpe AH. The B7 Family Revisited. Annu Rev Immunol (2005) 23:515–48. doi: 10.1146/annurev.immunol.23.021704.115611 15771580

[118] Xiao Y Yu S Zhu B Bedoret D Bu X Francisco LM. RGMb is a Novel Binding Partner for PD-L2 and its Engagement With PD-L2 Promotes Respiratory Tolerance. J Exp Med (2014) 211:943–59. doi: 10.1084/jem.20130790 PMC4010901 24752301

[119] Latchman Y Wood CR Chernova T Chaudhary D Borde M Chernova I. PD-L2 Is a Second Ligand for PD-1 and Inhibits T Cell Activation. Nat Immunol (2001) 2:261–8. doi: 10.1038/85330 11224527

[120] Ko EC Raben D Formenti SC. The Integration of Radiotherapy With Immunotherapy for the Treatment of Non-Small Cell Lung Cancer. Clin Cancer Res (2018) 24:5792–806. doi: 10.1158/1078-0432.CCR-17-3620 29945993

[121] Shaverdian N Lisberg AE Bornazyan K Veruttipong D Goldman JW Formenti SC. Previous Radiotherapy and the Clinical Activity and Toxicity of Pembrolizumab in the Treatment of Non-Small-Cell Lung Cancer: A Secondary Analysis of the KEYNOTE-001 Phase 1 Trial. Lancet Oncol (2017) 18:895–903. doi: 10.1016/S1470-2045(17)30380-7 28551359 PMC5538772

[122] Myers CJ Lu B. Decreased Survival After Combining Thoracic Irradiation and an Anti-PD-1 Antibody Correlated With Increased T-Cell Infiltration Into Cardiac and Lung Tissues. Int J Radiat Oncol Biol Phys (2017) 99:1129–36. doi: 10.1016/j.ijrobp.2017.06.2452 PMC5726785 29165283

[123] Zhao J Yorke ED Li L Kavanagh BD Li XA Das S. Simple Factors Associated With Radiation-Induced Lung Toxicity After Stereotactic Body Radiation Therapy of the Thorax: A Pooled Analysis of 88 Studies. Int J Radiat Oncol Biol Phys (2016) 95:1357–66. doi: 10.1016/j.ijrobp.2016.03.024 PMC5541363 27325482

[124] Oshima Y Tanimoto T Yuji K Tojo A. EGFR-TKI-Associated Interstitial Pneumonitis in Nivolumab-Treated Patients With Non-Small Cell Lung Cancer. JAMA Oncol (2018) 4:1112–5. doi: 10.1001/jamaoncol.2017.4526 PMC5885195 29327061

[125] Routy B Le Chatelier E Derosa L Duong CPM Alou MT Daillère R. Gut Microbiome Influences Efficacy of PD-1-Based Immunotherapy Against Epithelial Tumors. Science (2018) 359:91–7. doi: 10.1126/science.aan3706 29097494

[126] Jin Y Dong H Xia L Yang Y Zhu Y Shen Y. The Diversity of Gut Microbiome is Associated With Favorable Responses to Anti-Programmed Death 1 Immunotherapy in Chinese Patients With NSCLC. J Thorac Oncol (2019) 14:1378–89. doi: 10.1016/j.jtho.2019.04.007 31026576

[127] Chaput N Lepage P Coutzac C Soularue E Le Roux K Monot C. Baseline Gut Microbiota Predicts Clinical Response and Colitis in Metastatic Melanoma Patients Treated With Ipilimumab. Ann Oncol (2017) 28:1368–79. doi: 10.1093/annonc/mdx108 28368458

[128] Wang Y Wiesnoski DH Helmink BA Gopalakrishnan V Choi K Dupont HL. Fecal Microbiota Transplantation for Refractory Immune Checkpoint Inhibitor-Associated Colitis. Nat Med (2018) 24:1804–8. doi: 10.1038/s41591-018-0238-9 PMC6322556 30420754

[129] Hakozaki T Richard C Elkrief A Hosomi Y Benlaïfaoui M Mimpen I. The Gut Microbiome Associates With Immune Checkpoint Inhibition Outcomes in Patients With Advanced Non-Small Cell Lung Cancer. Cancer Immunol Res (2020) 8:1243–50. doi: 10.1158/2326-6066.CIR-20-0196 32847937

[130] Bingula R Filaire M Radosevic-Robin N Bey M Berthon JY Bernalier-Donadille A. Desired Turbulence? Gut-Lung Axis, Immunity, and Lung Cancer. J Oncol (2017) 2017:5035371. doi: 10.1155/2017/5035371 29075294 PMC5623803

[131] Zhang YB Liu SJ Hu ZD Zhou JX Wang YZ Fang B. Increased Th17 Activation and Gut Microbiota Diversity are Associated With Pembrolizumab-Triggered Tuberculosis. Cancer Immunol Immunother (2020) 69:2665–71. doi: 10.1007/s00262-020-02687-5 PMC11027455 32761425

[132] Gopalakrishnan V Spencer CN Nezi L Reuben A Andrews MC Karpinets TV. Gut Microbiome Modulates Response to Anti-PD-1 Immunotherapy in Melanoma Patients. Science (2018) 359:97–103. doi: 10.1126/science.aan4236 29097493 PMC5827966

[133] Sivan A Corrales L Hubert N Williams JB Aquino-Michaels K Earley ZM. Commensal Bifidobacterium Promotes Antitumor Immunity and Facilitates Anti-PD-L1 Efficacy. Science (2015) 350:1084–9. doi: 10.1126/science.aac4255 PMC4873287 26541606

[134] Zhang C Wang J Sun Z Cao Y Mu Z Ji X. Commensal Microbiota Contributes to Predicting the Response to Immune Checkpoint Inhibitors in non-Small-Cell Lung Cancer Patients. Cancer Sci (2021) 112:3005–17. doi: 10.1111/cas.14979 PMC8353904 34028936

[135] Marschner D Falk M Javorniczky NR Hanke-Muller K Rawluk J Schmitt-Graeff A. MicroRNA-146a Regulates Immune-Related Adverse Events Caused by Immune Checkpoint Inhibitors. JCI Insight (2020) 5:e132334. doi: 10.1172/jci.insight.132334 PMC7213806 32125286

[136] Thibult ML Mamessier E Gertner-Dardenne J Pastor S Just-Landi S Xerri L. PD-1 Is a Novel Regulator of Human B-Cell Activation. Int Immunol (2013) 25:129–37. doi: 10.1093/intimm/dxs098 23087177

[137] Garris CS Arlauckas SP Kohler RH Trefny MP Garren S Piot C. Successful Anti-PD-1 Cancer Immunotherapy Requires T Cell-Dendritic Cell Crosstalk Involving the Cytokines IFN-γ and IL-12. Immunity (2018) 49:1148–61. doi: 10.1016/j.immuni.2018.09.024 PMC6301092 30552023

[138] Sivori S Pende D Quatrini L Pietra G Della Chiesa M Vacca P. NK Cells and ILCs in Tumor Immunotherapy. Mol Aspects Med (2021) 80:100870. doi: 10.1016/j.mam.2020.100870 32800530

[139] Hsu J Hodgins JJ Marathe M Nicolai CJ Bourgeois-Daigneault MC Trevino TN. Contribution of NK Cells to Immunotherapy Mediated by PD-1/PD-L1 Blockade. J Clin Invest (2018) 128:4654–68. doi: 10.1172/JCI99317 PMC6159991 30198904

[140] Brahmer JR Lacchetti C Schneider BJ Atkins MB Brassil KJ Caterino JM. Management of Immune-Related Adverse Events in Patients Treated With Immune Checkpoint Inhibitor Therapy: American Society of Clinical Oncology Clinical Practice Guideline. J Clin Oncol (2018) 36:1714–68. doi: 10.1200/JCO.2017.77.6385 PMC6481621 29442540

[141] Spain L Diem S Larkin J. Management of Toxicities of Immune Checkpoint Inhibitors. Cancer Treat Rev (2016) 44:51–60. doi: 10.1016/j.ctrv.2016.02.001 26874776

[142] Howell M Lee R Bowyer S Fusi A Lorigan P. Optimal Management of Immune-Related Toxicities Associated With Checkpoint Inhibitors in Lung Cancer. Lung Cancer (2015) 88:117–23. doi: 10.1016/j.lungcan.2015.02.007 25776466

[143] Puzanov I Diab A Abdallah K Bingham CO 3rd Brogdon C Dadu R. Managing Toxicities Associated With Immune Checkpoint Inhibitors: Consensus Recommendations From the Society for Immunotherapy of Cancer (SITC) Toxicity Management Working Group. J Immunother Cancer (2017) 5:95. doi: 10.1186/s40425-017-0300-z 29162153 PMC5697162

[144] Naqash AR Yang LV Sanderlin EJ Atwell DC Walker PR. Interleukin-6 as One of the Potential Mediators of Immune-Related Adverse Events in Non-Small Cell Lung Cancer Patients Treated With Immune Checkpoint Blockade: Evidence From a Case Report. Acta Oncol (2018) 57:705–8. doi: 10.1080/0284186X.2017.1406668 29171332

[145] Stroud CR Hegde A Cherry C Naqash AR Sharma N Addepalli S. Tocilizumab for the Management of Immune Mediated Adverse Events Secondary to PD-1 Blockade. J Oncol Pharm Pract (2019) 25:551–7. doi: 10.1177/1078155217745144 29207939

[146] von Itzstein MS Khan S Gerber DE. Investigational Biomarkers for Checkpoint Inhibitor Immune-Related Adverse Event Prediction and Diagnosis. Clin Chem (2020) 66:779–93. doi: 10.1093/clinchem/hvaa081 PMC7259479 32363387

[147] Lin X Deng H Yang Y Wu J Qiu G Li S. Peripheral Blood Biomarkers for Early Diagnosis, Severity, and Prognosis of Checkpoint Inhibitor-Related Pneumonitis in Patients With Lung Cancer. Front Oncol (2021) 11:698832. doi: 10.3389/fonc.2021.698832 34327140 PMC8313853

[148] Schoenfeld JD Nishino M Severgnini M Manos M Mak RH Hodi FS. Pneumonitis Resulting From Radiation and Immune Checkpoint Blockade Illustrates Characteristic Clinical, Radiologic and Circulating Biomarker Features. J Immunother Cancer (2019) 7:112. doi: 10.1186/s40425-019-0583-3 31014385 PMC6480873

[149] Hoefsmit EP Rozeman EA Haanen J Blank CU. Susceptible Loci Associated With Autoimmune Disease as Potential Biomarkers for Checkpoint Inhibitor-Induced Immune-Related Adverse Events. ESMO Open (2019) 4:e000472. doi: 10.1136/esmoopen-2018-000472 31423333 PMC6677983

[150] Adam K Iuga A Tocheva AS Mor A. A Novel Mouse Model for Checkpoint Inhibitor-Induced Adverse Events. PloS One (2021) 16:e0246168. doi: 10.1371/journal.pone.0246168 33571254 PMC7877613

[151] Guerin MV Finisguerra V Van den Eynde BJ Bercovici N Trautmann A. Preclinical Murine Tumor Models: A Structural and Functional Perspective. Elife (2020) 9:e50740. doi: 10.7554/eLife.50740 31990272 PMC6986875

[152] Jia XH Geng LY Jiang PP Xu H Nan KJ Yao Y. The Biomarkers Related to Immune Related Adverse Events Caused by Immune Checkpoint Inhibitors. J Exp Clin Cancer Res (2020) 39:284. doi: 10.1186/s13046-020-01749-x 33317597 PMC7734811

[153] Matsukane R Watanabe H Minami H Hata K Suetsugu K Tsuji T. Continuous Monitoring of Neutrophils to Lymphocytes Ratio for Estimating the Onset, Severity, and Subsequent Prognosis of Immune Related Adverse Events. Sci Rep (2021) 11:1324. doi: 10.1038/s41598-020-79397-6 33446685 PMC7809015

[154] Egami S Kawazoe H Hashimoto H Uozumi R Arami T Sakiyama N. Peripheral Blood Biomarkers Predict Immune-Related Adverse Events in Non-Small Cell Lung Cancer Patients Treated With Pembrolizumab: A Multicenter Retrospective Study. J Cancer (2021) 12:2105–12. doi: 10.7150/jca.53242 PMC7974524 33754009

[155] Paz-Ares LG Ramalingam SS Ciuleanu TE Lee JS Urban L Caro RB. First-Line Nivolumab Plus Ipilimumab in Advanced Non-Small Cell Lung Cancer: 4-Year Outcomes From the Randomized, Open-Label, Phase 3 CheckMate 227 Part 1 Trial. J Thorac Oncol (2022) 17:289–308. doi: 10.1016/j.jtho.2021.09.010 34648948

[156] Chae YK Arya A Iams W Cruz MR Chandra S Choi J. Current Landscape and Future of Dual Anti-CTLA4 and PD-1/PD-L1 Blockade Immunotherapy in Cancer; Lessons Learned From Clinical Trials With Melanoma and Non-Small Cell Lung Cancer (NSCLC). J Immunother Cancer (2018) 6:39. doi: 10.1186/s40425-018-0349-3 29769148 PMC5956851

[157] Ramos-Casals M Brahmer JR Callahan MK Flores-Chavez A Keegan N Khamashta MA. Immune-Related Adverse Events of Checkpoint Inhibitors. Nat Rev Dis Primers (2020) 6:38. doi: 10.1038/s41572-020-0160-6 32382051 PMC9728094

[158] Kon E Benhar I. Immune Checkpoint Inhibitor Combinations: Current Efforts and Important Aspects for Success. Drug Resist Update (2019) 45:13–29. doi: 10.1016/j.drup.2019.07.004 31382144

[159] Srivastava S Furlan SN Jaeger-Ruckstuhl CA Sarvothama M Berger C Smythe KS. Immunogenic Chemotherapy Enhances Recruitment of CAR-T Cells to Lung Tumors and Improves Antitumor Efficacy When Combined With Checkpoint Blockade. Cancer Cell (2021) 39:193–208. doi: 10.1016/j.ccell.2020.11.005 33357452 PMC7878409

